# Electrochemical self-powered biosensors for autonomous diagnostics and monitoring: energy sources, recognition interfaces, and translational design rules

**DOI:** 10.3389/fbioe.2026.1967612

**Published:** 2026-10-29

**Authors:** Abdellatif Ait Lahcen, Gymama Slaughter

**Affiliations:** 1 Center for Bioelectronics, Old Dominion University, Norfolk, VA, United States; 2 Indian Institute of Technology Delhi–Abu Dhabi, Khalifa City, Abu Dhabi, United Arab Emirates; 3 Department of Electrical and Computer Engineering, Norfolk, VA, United States

**Keywords:** biofuel cells, electrochemical biosensors, enzymatic biofuel cells, microbial fuel cells, molecular recognition, point-of-care sensing, self-powered biosensors, wearable diagnostics

## Abstract

Electrochemical self-powered biosensors are autonomous platforms that convert biochemical, microbial, photochemical, or mechanical energy into diagnostic signals, reducing dependence on batteries and conventional potentiostats. Their compact, low-power operation makes them attractive for wearable monitoring, point-of-care testing, implantable devices, and environmental analysis. This review primarily focuses on intrinsically self-powered electrochemical platforms, including enzymatic and microbial fuel cells, organelle-based systems, abiotic devices, and sample-powered sensors, while selected TENG/PENG-based systems are discussed to illustrate how mechanically harvested energy can support electrochemical sensing. Particular emphasis is placed on molecular recognition interfaces including enzymes, microbial biofilms, organelles, antibodies, aptamers, nucleic acids, molecularly imprinted polymers, nanozymes, ion-selective membranes, and nanostructured electrodes that link energy generation with analytical specificity. We discuss how these interfaces regulate current, voltage, power, photocurrent, electron transfer, and charge storage, and highlight strategies for improving recognition efficiency, electron transfer, mass transport, antifouling performance, and stability. Key challenges include low power density, unstable biointerfaces, mediator leaching, biofouling, matrix effects, motion artifacts, scalability, biocompatibility, and limited standardization. Finally, we propose design principles for developing robust, selective, manufacturable, and clinically relevant electrochemical self-powered biosensors for next-generation autonomous diagnostics.

## 1 Introduction

Electrochemical biosensors are essential analytical platforms because they offer high sensitivity, fast response, low cost, miniaturization, and easy integration into portable and wearable formats (Gao et al., 2023; Wu et al., 2023). Since they emerged as practical analytical devices, biosensors have progressed beyond conventional glucose monitoring to support point-of-care diagnostics, personalized medicine, environmental analysis, food safety, and continuous health monitoring. Turner’s landmark review described biosensors as mature and influential analytical tools with broad applications in medicine, pharmaceutical analysis, food and process control, environmental monitoring, defense, and security (Turner, 2013). Building on this foundation, later reviews of wearable electrochemical sensors highlighted their value for continuous, real-time, non-invasive monitoring of clinically relevant electrolytes and metabolites in accessible biofluids, including sweat, saliva, tears, and interstitial fluid (Bandodkar and Wang, 2014; Gao et al., 2023).

Moreover, wearable and flexible electrochemical sensors are accelerating the shift from centralized testing to continuous, decentralized diagnostics (Wu et al., 2023). By analyzing sweat, saliva, tears, interstitial fluid, and other accessible biofluids, they enable real-time personalized monitoring beyond clinical settings. Recent reviews also highlight their role in disease monitoring, biomarker discovery, and personalized healthcare, especially when electrochemical detection is combined with soft materials, microfluidics, wireless communication, and data analytics (Chen et al., 2023; Duan et al., 2025). Despite these advantages, many electrochemical biosensors still require external power supplies, potentiostats, batteries, or signal-processing electronics (del Campo, 2023). This dependence hinders miniaturization, long-term operation, cost reduction, and sustainability, particularly in wearable, implantable, point-of-care, and remote diagnostics where autonomous, continuous sensing with minimal user intervention is needed (Li et al., 2025). In this context, self-powered biosensors have therefore emerged as promising next-generation diagnostic platforms. They harvest energy from the analyte, sample chemistry, body motion, light, heat, or other environmental sources to support more autonomous sensing architectures (Li et al., 2023).

Electrochemical self-powered biosensors convert biochemical, microbial, photochemical, or mechanical energy into electrical signals related to target concentration, reducing or eliminating external bias during sensing (Gao Y. et al., 2025). Biofuel cell-based biosensors are the most established examples. The resulting current, voltage, or power can be correlated with analyte concentration, making the biofuel cell both the energy source and sensing platform. Grattieri and Minteer identified enzymatic, organelle-based, and microbial fuel-cell biosensors as key self-powered systems, while Hao et al. reviewed biofuel-cell-based platforms for clinical, environmental, and industrial applications Hao et al., 2020). Consequently, electrochemical self-powered biosensors differ from general energy-harvesting biosensors because electrochemical reactions can simultaneously provide energy conversion, molecular recognition, and analytical transduction (Yuen et al., 2019). Unlike conventional electrochemical biosensors that require an external potential, these systems rely on intrinsic potentials generated by paired anodic and cathodic reactions. This coupling creates trade-offs among sensitivity, selectivity, electron transfer, mass transport, and power output (Fredj et al., 2025). Therefore, they must be assessed using both analytical metrics and self-powered-specific metrics.

This review focuses on electrochemical self-powered biosensors for autonomous diagnostics, with biofuel cell-based systems as the core platform because they directly integrate energy generation and sensing. The scope encompasses enzymatic and microbial fuel cells, organelle-based systems, abiotic biosensors, and sample-powered devices, including platforms that employ nanozymes and other engineered catalytic interfaces. Because relatively few TENG/PENG studies incorporate a dedicated electrochemical sensing module with specific biochemical recognition, selected examples are included to clarify their relationship to electrochemical self-powered biosensing. These systems are classified according to their architecture as power-assisted, self-charging, or hybrid platforms when the nanogenerator powers an electrochemical sensing module or is coupled to a biochemical recognition interface, rather than being treated as intrinsically self-powered electrochemical biosensors.

Unlike reviews that mainly summarize recent advances (Chen et al., 2019; Hao et al., 2020; Galstyan et al., 2024; Gupta and Krasnoslobodtsev, 2024; Li et al., 2025; Grattieri and Minteer, 2018; Slaughter and Kulkarni, 2015) this review proposes a design-rule framework linking energy source, recognition interface, signal generation, analytical performance, device architecture, and translational readiness. It clarifies what qualifies as a truly self-powered electrochemical biosensor, how energy is converted into analytical signals, how power output is evaluated alongside biosensing metrics, and which barriers must be overcome for wearable, implantable, and point-of-care diagnostics. Table 1 summarizes the existing review landscape and highlights the specific gaps addressed by the present review.

**TABLE 1:** Existing review landscape and positioning of the present review.

Existing review direction	Main focus	Remaining gap	How our review differs	Ref.
General self-powered biosensors	Enzymatic, organelle-based, and microbial fuel-cell biosensors	Earlier review; less focus on recent wearable, MIP, nanozyme, photoelectrochemical, and translation issues	Updates the field and adds a diagnostic design-rule framework	(Grattieri and Minteer, 2018)
Biofuel-cell-based self-powered biosensors	Enzymatic and microbial fuel-cell biosensors	Mainly organized by BFC construction and applications	Links BFC operation to molecular recognition and analytical benchmarking	(Hao et al., 2020)
Enzymatic biofuel-cell biosensors	EBFCs for self-powered, wearable, and implantable biosensing	Strong enzyme focus; less attention to artificial receptors and non-fuel biomarkers	Expands to MIPs, nanozymes, aptamers, antibodies, and hybrid recognition	(Gupta and Krasnoslobodtsev, 2024) (Fredj et al., 2025)
Glucose-specific enzymatic biofuel cells	EBFCs as implantable power sources; DET vs. MET mechanisms; with *in vivo* case studies across multiple organisms and applications	Glucose-only scope; treats the biofuel cell as a power source for downstream electronics rather than a self-contained sensing platform	Extends beyond glucose-specific power generation to the full platform taxonomy and recognition-interface taxonomy	(Slaughter and Kulkarni, 2015)
Disease-oriented self-powered electrochemical biosensors	Early disease diagnosis using self-powered electrochemical biosensors	Application-centered; less emphasis on general design rules and benchmarking	Proposes broader energy–signal coupling and translation framework	(Galstyan et al., 2024)
Wearable self-powered biosensors	Wearable power systems, energy harvesters, sweat biosensors	Strong wearable focus; may include non-electrochemical systems	Focuses specifically on electrochemical diagnostic mechanisms	(Song et al., 2021; Li et al., 2023)
Microbial fuel-cell SPBs	Environmental and microbial fuel-cell sensing	Mostly environmental and microbial systems	Places MFCs within a broader electrochemical diagnostic framework	(Liu et al., 2024)

Building on this positioning, the review also examines the specific recognition and interface components that connect these platforms to analytical performance, including enzymes, microbial biofilms, organelles, antibodies, aptamers, DNA probes, molecularly imprinted polymers, nanozymes, ion-selective membranes, conductive and redox polymers, hydrogels, antifouling layers, and nanostructured electrodes. Emphasis is placed on how these interfaces convert target recognition into self-generated outputs such as current, voltage, power, photocurrent, open-circuit voltage, short-circuit current, or stored charge, and on the power management, interface stability, real-sample reliability, scalable manufacturing, biocompatibility, sustainability, and standardized-reporting barriers that determine translational readiness.

The literature was collected from Scopus, PubMed, and Google Scholar, focusing primarily on publications from January 2014 to May 2026 while also including foundational studies on biofuel cells, microbial processes, organelle-based systems, and self-powered sensing. Search terms included combinations of keywords such as “self-powered electrochemical biosensor,” “biofuel cell biosensor,” “microbial fuel cell biosensor,” “wearable biosensor,” and related terms describing energy-generation and molecular-recognition mechanisms. Studies were selected when they incorporated an electrochemical sensing mechanism, clearly identified the energy source and recognition element, and provided sufficient information to evaluate device architecture or analytical performance. Key studies were selected to represent diverse platform types, recognition interfaces, sample matrices, applications, and stages of technological development. Performance comparisons excluded mechanically driven nanogenerators that lacked biochemical recognition or a dedicated electrochemical sensing mechanism. Review articles were used to clarify terminology, identify foundational and relevant studies, and contextualize technological maturity. This approach constitutes a structured narrative review rather than a systematic review or meta-analysis.

## 2 Conceptual classification of electrochemical self-powered biosensors

The rapid growth of self-powered biosensing has produced many device types with different energy sources, signal-generation mechanisms, recognition strategies, and integration levels. The term “self-powered biosensor” has been applied to enzymatic and microbial biofuel cell biosensors, sample- or fuel-cell-powered electrochemical sensors, self-powered photoelectrochemical biosensors, nanogenerator-assisted platforms, and hybrids with capacitors or other energy-storage units (Zeng et al., 2022). Although all aim to reduce reliance on external power supplies, batteries, or potentiostats, they operate by different mechanisms. In some systems, the sample or target directly supplies the chemical energy for signal generation; in others, an energy harvester powers a separate sensing interface. Electrochemical self-powered biosensors are distinct because electrochemical reactions can couple energy conversion, molecular recognition, and signal transduction. These systems function simultaneously as both the power source and the biosensor, as demonstrated by enzymatic and microbial fuel cell-based platforms for toxicity, COD, BOD, and assimilable organic carbon detection (Slaughter and Kulkarni, 2015; Hao et al., 2020). Sailapu and Menon further defined sample-powered electrochemical sensors as systems that use chemical species in the analyzed liquid as the sole energy source, helping distinguish them from broader energy-harvesting devices (Sailapu and Menon, 2022). Clear classification is therefore needed to avoid ambiguity and compare both analytical performance and self-powered operation.

To establish conceptual consistency, we classify the systems considered in this review into two scope-based groups (Scheme 1). (A) The primary group comprises intrinsically self-powered electrochemical biosensors in which biochemical or electrochemical processes generate analyte-dependent signals without externally applied bias. This group includes enzymatic biofuel cells, microbial fuel cells, organelle-based systems, abiotic systems, and sample-powered devices. (B) A secondary group comprises selected TENG/PENG-assisted electrochemical systems in which mechanical energy is harvested separately from molecular recognition. Depending on their architecture, these systems are classified as power-assisted, self-charging, or hybrid platforms. Only TENG/PENG systems incorporating both a defined electrochemical sensing mechanism and a biochemical recognition interface are considered. Zero-bias photoelectrochemical biosensors are not treated as a separate platform category within the scope of this review; selected examples are discussed only where they provide relevant insight into recognition-interface or energy-signal coupling mechanisms.

**SCHEME 1:**
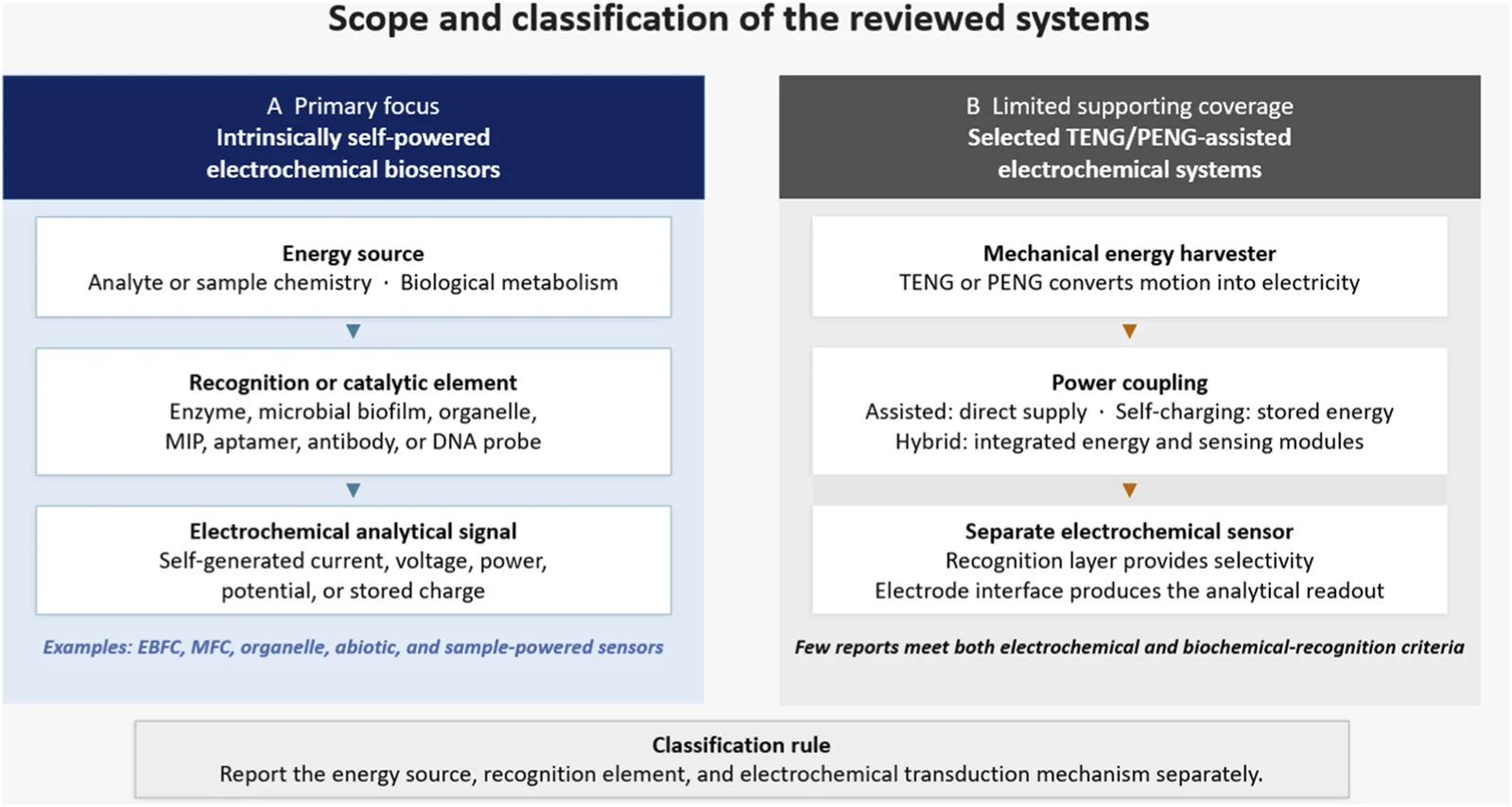
Conceptual classification of electrochemical self-powered biosensors based on the relationship among energy source, molecular recognition, and electrochemical transduction. The primary scope comprises intrinsically self-powered electrochemical biosensors, while selected TENG/PENG-based power-assisted, self-charging, and hybrid architectures are included for comparison.

Across these classifications, each platform can be described by three interconnected functions: an energy source that drives the self-powered process, a recognition interface that provides molecular selectivity or target-dependent gating, and a transduction mechanism that converts recognition into a measurable analytical signal. These functions need not correspond to separate physical components. In enzymatic biofuel cells, for example, an enzyme may simultaneously recognize the fuel and catalyze the reaction responsible for power generation. In affinity-based systems, antibodies, aptamers, nucleic acid probes, molecularly imprinted polymers, or related receptors primarily provide molecular recognition, while target binding or hybridization alters interfacial processes that are subsequently converted into measurable electrical signals. This energy-recognition-transduction framework provides a common basis for comparing otherwise diverse self-powered electrochemical biosensors.

Unless explicitly linked to a cited standard, numerical thresholds presented in the Design Rules represent practical benchmarks proposed in this review to promote more consistent and comparable validation. They should not be interpreted as formal standards, mandatory acceptance criteria, or universally established thresholds. Their applicability should instead be considered in relation to device architecture, intended use, sample matrix, and stage of technological development.

### 2.1 Enzymatic biofuel cell-based self-powered biosensors

Enzymatic biofuel cell-based self-powered biosensors are among the most established platforms in electrochemical self-powered biosensing. An enzymatic biofuel cell (EBFC) converts biochemical energy from relevant fuels into electrical energy using isolated redox enzymes. Fuel oxidation at the bioanode and oxidant reduction at the biocathode generate a potential difference that drives electron flow, producing measurable current, voltage, open-circuit voltage, short-circuit current, or power output (Slaughter et al., 2016). In biosensing, this output correlates with analyte concentration or powers a coupled readout (Gupta and Krasnoslobodtsev, 2024). This dual role defines self-powered electrochemical biosensing, where biofuel cells act as both the energy source and sensing platform (Slaughter et al., 2016; Yi et al., 2024). A defining feature of EBFC-based self-powered biosensors is that enzymes serve as both recognition and catalytic elements. Common anodic enzymes include glucose oxidase, glucose dehydrogenase, lactate oxidase, lactate dehydrogenase, alcohol dehydrogenase, alcohol oxidase, and cholesterol oxidase, while bilirubin oxidase and laccase are commonly used for cathodic oxygen reduction. These enzymes provide substrate selectivity and bioelectrocatalytic conversion under mild physiological conditions.

An EBFC operates similarly to a conventional fuel cell: a fuel is enzymatically oxidized at the anode, releasing electrons that travel through an external circuit to the cathode, where an oxidant, typically O_2_, is reduced (Figure 1). The resulting potential difference drives current flow, allowing enzyme-catalyzed substrate oxidation to generate electrical power (Barelli et al., 2021). In the simplest EBFC sensing format, the target analyte also acts as the fuel. Its concentration controls the rate of anodic bioelectrocatalytic oxidation and therefore modulates the generated current, voltage, or power. This approach has been widely used for metabolite detection. Examples include reagentless self-powered cholesterol sensing in human plasma (Sekretaryova et al., 2014), glucose detection in patient plasma (Slaughter and Kulkarni, 2019), ethanol detection using a β-NAD^+^-dependent alcohol dehydrogenase bioanode and oxygen-reducing biocathode (Ruff et al., 2017), printed-circuit-board glucose/O_2_ fuel cells for self-powered glucose sensing (Gonzalez-Solino et al., 2021), sensitive EBFC-based glucose detection (Chansaenpak et al., 2021), mediator-free glucose sensing using a hybrid glucose/MnO_2_ EBFC (Hao et al., 2021), and disposable glucose sensing based on short-circuit current readout (Morshed et al., 2023). Collectively, these characteristics make EBFCs especially attractive for wearable and implantable biosensing because they can use endogenous fuels such as glucose, lactate, ethanol, and other metabolites in biological fluids (Gupta and Krasnoslobodtsev, 2024; Fredj et al., 2025).

**FIGURE 1:**
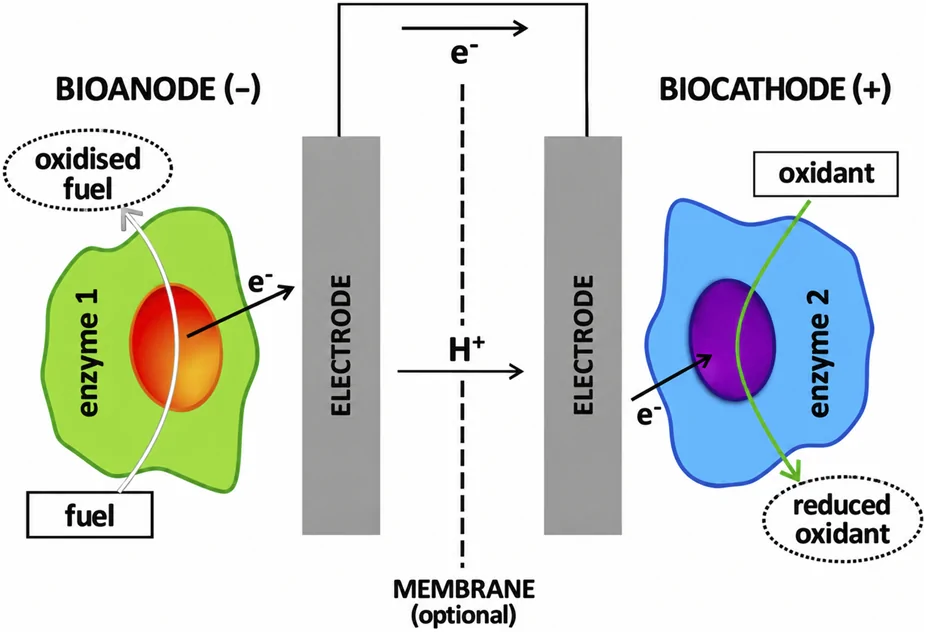
Operating principle of an enzymatic biofuel cell, showing enzymatic fuel oxidation at the bioanode, electron transfer through the external circuit, and oxidant reduction at the biocathode (Barelli et al., 2021).

Wearable EBFC biosensors apply this principle to on-body monitoring, where sweat and other accessible biofluids provide both the sample and the fuel. Stretchable textile biofuel cells have demonstrated wearable self-powered sensing under mechanical deformation (Jeerapan et al., 2016), while epidermal EBFC sensors enabled *in situ* sweat glucose and lactate monitoring (Huang et al., 2022). More recently, a lactate biofuel-cell sensor supported continuous sweat lactate monitoring for sports and medical applications (Shitanda et al., 2025). Together, these studies show the value of EBFCs for wearable diagnostics when the monitored metabolite also serves as fuel. EBFCs can also support indirect sensing, where non-fuel targets modulate the fuel-cell output.

The performance of these systems depends on enzyme kinetics and stability, immobilization, electron transfer, fuel and oxygen transport, electrode architecture, and sample composition. These factors are interdependent. For example, strategies that improve enzyme stability or reduce biofouling may restrict mass transport and consequently decrease electrical output. EBFC design therefore requires co-optimization of the recognition interface, electrode architecture, mass transport, and operating environment rather than maximizing any single parameter. Broader challenges related to stability, biofouling, matrix effects, and translation across self-powered platforms are discussedin Sections 3.11 and 4 (Gupta and Krasnoslobodtsev, 2024; Fredj et al., 2025).

Accordingly, EBFC-based self-powered biosensors are therefore assessed using both analytical and biofuel-cell metrics. Analytical evaluation includes sensitivity, selectivity, linear range, detection limit, response time, reproducibility, recovery, and operational stability, whereas biofuel-cell evaluation includes open-circuit voltage, short-circuit current, current density, maximum power density, internal resistance, power stability, charging ability, and compatibility with low-power readout electronics. This dual assessment is essential because high power output does not guarantee selective or sensitive detection, and high sensitivity does not ensure sufficient power for autonomous operation. Progress toward wearable, implantable, and point-of-care applications will require co-optimization of the enzyme interface, electrode architecture, fuel-cell configuration, and readout strategy and should be guided by the following design rules in Box 1.

BOX 1 **Design Rules for EBFCs.**

1. Maximize direct or mediated electron transfer between the enzyme and the electrode.
2. Stabilize the recognition interface against enzyme denaturation, leaching, and mediator loss.
3. Report analytical and energetic performance together. At minimum, report an appropriate analytical detection metric and a quantitative measure of self-powered output, such as maximum power density, current density, or open-circuit voltage, as appropriate to the device architecture. Neither analytical nor energetic performance alone is sufficient to characterize a self-powered biosensor.
4. Design for the endogenous fuel present in the target biofluid, not an idealized substrate.
5. Validate performance in the intended biological matrix. For devices targeting a specific biofluid, evaluate sufficient independent samples to characterize matrix-dependent variability and, where appropriate, compare results with an established reference method such as HPLC or a validated commercial assay. For platforms intended for operation across multiple biofluids, validation should include each claimed matrix.

### 2.2 Microbial fuel cell-based self-powered biosensors

Microbial fuel cell-based self-powered biosensors are the second major subgroup of biofuel cell-based biosensors. Unlike enzymatic systems, which use isolated enzymes, MFCs use living microorganisms or electroactive biofilms to convert biodegradable organic matter into electrical energy (Palmore and Whitesides, 1994). Microorganisms oxidize organic substrates at the anode and transfer electrons directly, through conductive pili or outer-membrane redox proteins, or indirectly via soluble mediators. These electrons flow to the cathode, where oxygen or another terminal acceptor is reduced. Because the output reflects microbial metabolism, substrate availability, and toxic inhibition, MFCs are useful self-powered biosensors for water-quality and environmental monitoring (Kumar et al., 2022). Recent reviews use “microbial fuel cell-based self-powered biosensors” to describe MFC devices whose current, voltage, or power output serves directly as the sensing signal. Hao et al. identified them as a major biofuel cell-based self-powered biosensor class for toxicity, COD, BOD, and AOC detection (Hao et al., 2020). Xue et al. further classified their sensing mechanisms into substrate-effect and microbial-activity-effect modes, distinguishing responses to biodegradable substrates from responses to toxic or inhibitory compounds (Xue et al., 2024).

Figure 2 shows the operating principle of an MFC-based biosensor, where electroactive microorganisms form an anodic biofilm that acts as the bioreceptor (Cui et al., 2019). When biodegradable substrate is present, microbial metabolism generates electrons that are transferred to the anode and then pass through an external circuit to the cathode, producing a measurable current signal.

**FIGURE 2:**
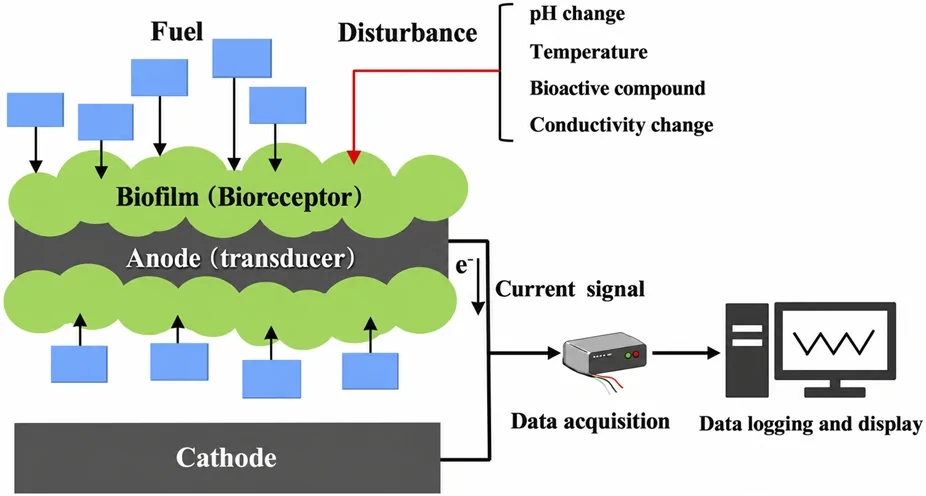
Operating principle of a microbial fuel-cell biosensor. Microbial metabolism generates electrons at the bioanode, and changes in the resulting electrical output are used for analytical monitoring. Reproduced/adapted from (Cui et al., 2019) with permission.

The MFC electrical output depends on the concentration and biodegradability of available organic matter, making it useful for BOD monitoring. Environmental factors such as pH, temperature, toxic or bioactive compounds, and conductivity also affect microbial activity and electron-transfer efficiency, thereby changing the electrical current response. This allows MFC biosensors to track variations in sample composition or environmental conditions. The electrical current signal is collected by a data-acquisition unit and transmitted to a logging and display system for real-time monitoring. Thus, an MFC biosensor combines microbial recognition in the biofilm, electrochemical transduction at the anode, and electronic recording for continuous analysis.

In substrate-effect MFC biosensors, the electrical signal reflects the amount of biodegradable organic matter. Higher concentrations of oxidizable compounds generally increase microbial metabolism and extracellular electron transfer, producing higher current or voltage within a suitable range. This principle is widely used for BOD and COD estimation in water and wastewater. BOD indicates the oxygen needed for microbial degradation of biodegradable organic matter, whereas COD reflects the oxygen required for chemical oxidation of organic and inorganic reducing substances. MFC-based BOD/COD biosensors are attractive because they provide faster, more continuous measurements than conventional laboratory methods while operating as self-powered bioelectrochemical systems. Yao et al. reviewed MFC-based organic-matter sensors and highlighted device configuration, operating conditions, and structural optimization as key performance factors (Yao et al., 2023). Tardy et al. further demonstrated an MFC biosensor for biodegradable organic matter in domestic wastewater, supporting its practical use for organic-load monitoring (Tardy et al., 2021).

In microbial-activity-effect MFC biosensors, the analyte changes microbial metabolism, viability, or electron-transfer activity. Toxicants such as heavy metals, antibiotics, phenols, pesticides, and other pollutants can inhibit microbial activity and lower the electrical current or voltage output. Zhou et al. reviewed MFC-based toxicity biosensors for monitoring heavy metals, including copper, chromium, and zinc, as well as organic toxicants such as p-nitrophenol, formaldehyde, and levofloxacin (Zhou et al., 2017). This inhibition-based mode is valuable for early-warning water-quality monitoring because output decreases can indicate toxic exposure. However, the response is often nonspecific and may require selective recognition or complementary analysis to identify the toxicant.

Beyond inhibition-based toxicity sensing, MFC-based self-powered biosensors have also been explored for assimilable organic carbon (AOC) monitoring, where the electrical response reflects microbial substrate utilization rather than toxic inhibition. AOC is the fraction of organic carbon readily consumed by microorganisms and is an important indicator of microbial regrowth potential in drinking-water distribution systems. Because MFC output is directly linked to microbial metabolism and substrate use, these systems are well suited to long-term autonomous AOC monitoring. Hao et al. identified AOC detection as an important application of MFC-based self-powered biosensors, particularly because AOC provides information not only about organic contamination but also about the potential for microbial regrowth and drinking-water instability (Hao et al., 2020).

A major strength of MFC-based self-powered biosensors is their ability to operate in complex environmental matrices while generating their own electrochemical signal. Unlike enzymatic biosensors, which often require purified enzymes and controlled immobilization conditions, MFC biosensors use living microbial communities that can adapt to real water and wastewater samples (Ma et al., 2022). This makes them useful for online monitoring, wastewater treatment plants, river-water surveillance, industrial effluents, and early-warning toxicity systems. Olias et al. emphasized response time, baseline recovery, electrode/bioreceptor ratio, flow conditions, and long-term stability as key factors for in-field water-quality monitoring (Olias and Di Lorenzo, 2021). However, the use of living microbial communities also introduces biological and environmental variability. MFC performance depends on microbial composition, biofilm maturity, pH, temperature, dissolved oxygen, nutrient availability, hydraulic conditions, and electrode architecture. These factors influence response time, baseline stability, selectivity, and reproducibility, requiring calibration and validation under conditions representative of the intended application (Cui et al., 2019).

These characteristics distinguish MFCs from EBFCs in both recognition and intended application. EBFCs typically provide molecular selectivity through enzyme-substrate recognition, whereas MFCs respond more broadly to microbial substrate utilization, metabolic activity, or toxic inhibition. MFCs are therefore particularly suited to complex environmental matrices, while EBFCs are advantageous when selective detection of a defined enzymatic substrate is required. Neither architecture is universally superior; platform selection should instead reflect the target, matrix, operating environment, and required analytical performance.

For electrochemical self-powered biosensor development, MFC systems are assessed using both environmental-sensing and bioelectrochemical metrics. Analytical metrics include sensitivity, linear range, response and recovery time, reproducibility, long-term stability, matrix tolerance, and agreement with standard BOD, COD, or toxicity assays. Bioelectrochemical metrics include open-circuit voltage, current density, power density, coulombic efficiency, internal resistance, baseline stability, and recovery after toxicant exposure. Single microbial strains may improve selectivity and reproducibility, whereas mixed consortia can provide broader substrate tolerance and greater robustness in wastewater (Zhou et al., 2017). MFC-based self-powered biosensors are more mature for environmental monitoring than clinical diagnostics, but their integration of microbial recognition, electrochemical transduction, and autonomous power generation makes them an important class of electrochemical self-powered biosensors and the following design rules could be applied to their development in Box 2.

BOX 2 **Design Rules for MFCs.**

1. Match microbial consortium choice (single strain vs. mixed) to the required balance of selectivity and robustness.
2. Optimize biofilm maturation, electrode/bioreceptor ratio, and flow conditions for reliable baseline recovery.
3. Benchmark analytical performance against an appropriate established reference method for the intended application, such as standard BOD, COD, AOC, or toxicity assays, while also reporting relevant bioelectrochemical output metrics.
4. Account for pH, temperature, and dissolved oxygen as calibration variables, not just noise sources.
5. Validate long-term operation in matrices representative of the intended application. Environmental MFC biosensors should progress from synthetic media to real wastewater or natural-water samples and report baseline drift, operational stability, response and recovery behavior, and performance over a duration appropriate to the intended monitoring application.

### 2.3 Organelle-based biofuel cell self-powered biosensors

Organelle-based biofuel-cell biosensors are a less explored but conceptually important subgroup of self-powered biosensors. Unlike enzymatic systems, which use isolated enzymes, or microbial systems, which use whole cells or biofilms, they rely on functional subcellular organelles as bioelectrocatalytic units. These organelles contain organized redox pathways, membrane-bound electron-transport chains, and compartmentalized biochemical machinery that can be coupled to electrodes for energy conversion and sensing. Grattieri and Minteer classified organelle-based biofuel cells alongside enzymatic and microbial platforms as major biofuel-cell systems relevant to self-powered biosensing (Grattieri and Minteer, 2018). The main organelles used in this context are mitochondria and photosynthetic structures, including chloroplasts, thylakoid membranes, photosystem I, and photosystem II. Mitochondria are attractive because their respiratory electron-transport chain can oxidize metabolic substrates such as pyruvate or NADH-linked fuels. Arechederra and Minteer demonstrated this concept by immobilizing mitochondria on carbon paper electrodes, where they oxidized pyruvate and acted as the bioanode of a biofuel cell (Arechederra and Minteer, 2008). This study showed that subcellular respiratory machinery can serve as a catalytic biofuel-cell component.

Figure 3 illustrates a thylakoid-based organelle self-powered biosensing system (Sarode and Slaughter, 2025b). Here, thylakoid membranes act as light-driven bioelectrocatalytic units, using Photosystem I and II to absorb light and generate electron flow. The electrons are collected at the anode and pass through an external circuit to the cathode, producing current or voltage as the sensing signal. Changes in thylakoid activity caused by analytes, inhibitors, or environmental factors alter electron transfer and produce a measurable response. Nanomaterials are used to support membrane immobilization and increase surface area, while redox mediators improve electron transfer when direct electrode communication is limited.

**FIGURE 3:**
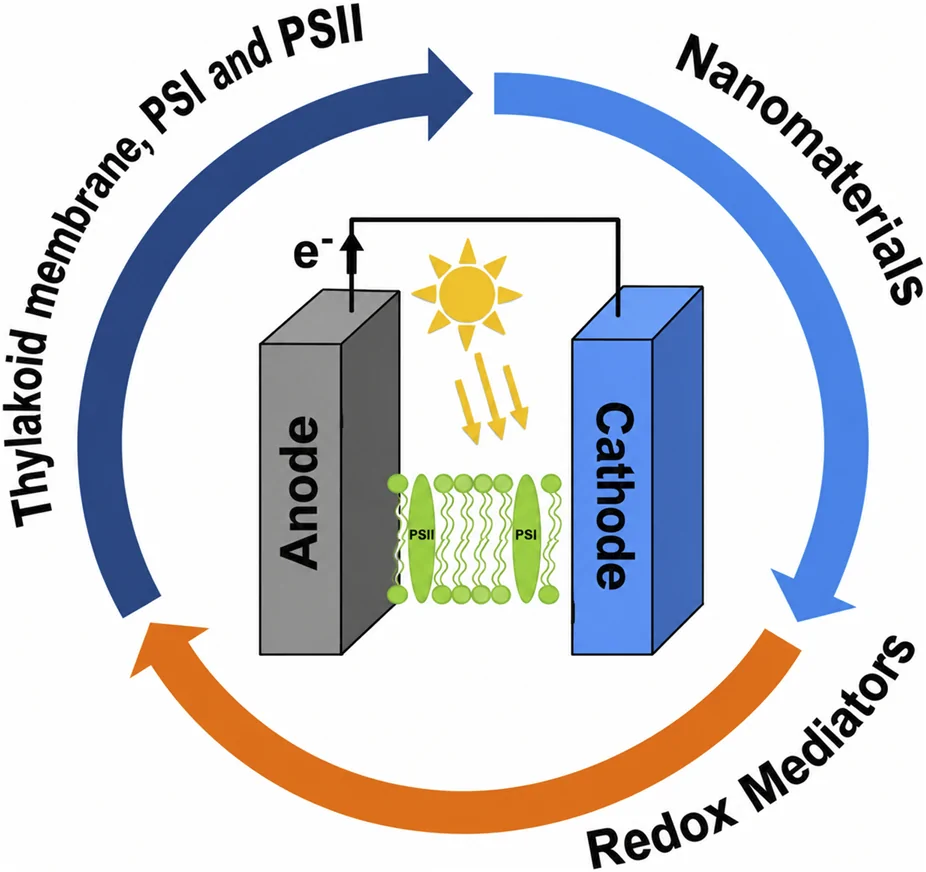
General principle of a thylakoid-based organelle self-powered biosensing system. Adapted from (Sarode and Slaughter, 2025b) with permission.

Photosynthetic organelles and organelle-derived membranes offer a complementary route by converting light into electrochemical energy through natural photosynthetic electron-transfer pathways. Thylakoid membranes, found in chloroplasts and cyanobacteria, contain the photosynthetic electron-transport chain and photosystems responsible for light-driven charge separation. In thylakoid-based biofuel cells, illumination generates excited electrons that are extracted at an electrode either directly or through redox mediators. Ryu et al. showed that thylakoid-deposited micropillar electrodes improved direct electron extraction by strengthening the coupling between photosynthetic membranes and solid electrodes (Ryu et al., 2018). Masi et al. later developed a photobiofuel cell using micro/nanostructured electrodes, a thylakoid-functionalized anode, and an oxygen-reducing biocathode to generate power under illumination (Masi et al., 2020). Together, these studies highlight the potential of photosynthetic organelle-derived systems as light-driven bioelectrochemical power sources.

For biosensing, organelle-based biofuel cells are valuable because organelles provide complex biological responses that are difficult to reproduce with isolated enzymes. Mitochondria respond to substrates, respiratory inhibitors, oxidative stress, and compounds that disrupt electron transport or membrane potential (Cui et al., 2019) making mitochondria-based electrodes relevant for monitoring metabolic activity, mitochondrial toxicity, and respiratory-chain inhibition. Similarly, thylakoid membranes and other photosynthetic organelles respond to light intensity, photosynthetic inhibitors, herbicides, redox mediators, and environmental stressors that affect photosystem activity or electron transport (Masi et al., 2020). These properties support their use in toxicity testing, environmental monitoring, photosynthetic inhibitor detection, and biohybrid energy-sensing platforms.

However, organelle-based self-powered biosensors remain less developed than enzymatic and microbial fuel cell biosensors. Most mitochondrial and thylakoid biofuel-cell studies focus on energy conversion, photocurrent generation, or electrode-interface design rather than validated analytical sensing. Practical limitations include complex organelle isolation, activity loss outside the native cellular environment, membrane fragility, short operational lifetime, inefficient electron transfer, and difficulty maintaining physiological conditions (Gu et al., 2022). Photosynthetic systems face additional challenges, including light dependence, photodamage, mediator instability, photosystem orientation, and weak contact between biological membranes and electrodes.

Accordingly, organelle-based biofuel-cell biosensors should be regarded as an emerging and mechanistically valuable class rather than a mature diagnostic technology. Their primary contribution is demonstrating that biological energy-conversion modules, such as mitochondria and thylakoid membranes, can be integrated with electrodes to generate self-powered electrochemical signals. Although their application to point-of-care diagnostics remains limited, these systems provide an important link among bioelectrocatalysis, cellular bioenergetics, photosynthetic bioelectrochemistry, and self-powered sensing. Future development will require improved organelle stabilization, more efficient direct or mediated electron transfer, selective recognition mechanisms, and analytical validation in realistic samples. Table 2 compares representative enzymatic, microbial, organelle-based, and biomimetic catalytic self-powered biosensors according to their biorecognition interfaces, energy sources, signal-output mechanisms, and analytical performance. Based on the limitations and opportunities identified above, the following design rules are proposed for organelle-based systems in Box 3.

BOX 3 **Design rules for organelle-based systems.**

1. Preserve organelle integrity and native electron-transfer chains during isolation and electrode immobilization.
2. Use nanomaterials or redox mediators to strengthen electrical coupling between the organelle and the electrode.
3. For photosynthetic organelles, control illumination intensity and duration to limit photodamage.
4. Benchmark against an appropriate non-organelle bioelectrochemical platform under matched conditions before claiming superior analytical or energetic performance.
5. Evaluate batch-to-batch reproducibility using independently prepared organelle isolations and report variability in analytical and, where applicable, bioelectrochemical output. As an author-proposed benchmark rather than an established standard, at least three independent organelle-isolation batches are recommended for assessing batch-to-batch variability.

**TABLE 2:** Representative self-powered biosensors based on enzymatic, microbial, organelle, and biomimetic catalytic interfaces.

Platform	Biorecognition/catalytic interface	Target/application	Fuel/energy source	Electrode/interface materials	Output	Sample matrix/validation
Enzymatic biofuel cell (Sekretaryova et al., 2014)	Cholesterol oxidase-based bioanode	Cholesterol	Cholesterol/O_2_	Enzyme-modified bioanode and enzymatic biocathode	Current/power	Buffer/model sample
Wearable enzymatic biofuel cell (Jeerapan et al., 2016)	Lactate oxidase	Sweat lactate	Lactate/O_2_	Stretchable textile bioelectrodes	Current/power	Human sweat/on-body
Enzymatic biofuel cell (Ruff et al., 2017)	Alcohol dehydrogenase-based bioanode	Ethanol	Ethanol/O_2_	Redox-polymer-wired enzymatic electrodes	Current/power	Buffer/model sample
Glucose/O_2_ enzymatic biofuel cell (Gonzalez-Solino et al., 2021)	Glucose oxidase and bilirubin oxidase	Glucose	Glucose/O_2_	Porous Au electrodes with Os-redox-polymer wiring	Current/power	Buffer/Saliva
Hybrid enzymatic–abiotic fuel cell (Hao et al., 2021)	Glucose oxidase bioanode	Glucose	Glucose/Mn O_2_	Enzymatic anode and MnO_2_ cathode	Current/power	Buffer/Grape juice/Human serum
Epidermal self-powered sensor (Huang et al., 2022)	Glucose oxidase or lactate oxidase	Sweat glucose/lactate	Sweat metabolites/O_2_	Flexible enzyme-functionalized electrodes	Current/power	Artificial/human sweat
Microbial fuel-cell biosensor (Ma et al., 2022)	Electroactive microbial biofilm	BOD/organic matter	Wastewater organics	Biofilm anode and oxygen-reducing cathode	Voltage/current	Wastewater
Disposable enzymatic biofuel cell (Morshed et al., 2023)	Glucose-oxidizing enzyme interface	Glucose	Glucose/O_2_	Disposable enzyme-modified electrodes	Short-circuit current	Glucose sample/model matrix
Photobiofuel cell (Masi et al., 2020)	Photosynthetic thylakoid membrane	Light-driven bioelectrocatalysis	Light/water	Micro/nanostructured thylakoid electrodes	Photocurrent/power	Buffer/model system
Organelle photo-bioelectrochemical cell (Sarode and Slaughter, 2025a)	Thylakoid membrane	Light-driven output	Light/water	PEDOT/thylakoid-modified laser-induced graphene	Photocurrent/power	Buffer
Wearable enzymatic biofuel cell (Shitanda et al., 2025)	Lactate oxidase-based interface	Sweat lactate	Lactate/O_2_	Wearable enzyme-modified electrodes	Current/power	Human sweat/on-body
Non-enzymatic biofuel cell (Lei et al., 2025)	AuNC/PdNC and Fe_3_C@C–Fe catalytic interfaces	Glucose	Glucose/O_2_	Au nanocluster/Pd nanocube anode and Fe single-atom cathode	Current/power	Biological sample

### 2.4 Power-assisted, self-charging, and hybrid platforms for electrochemical biosensing

The platforms considered so far, generate their electrical signal directly from a biochemical or biological energy source, so that recognition and power generation occur in the same reaction. The remaining platform classes take a different route: they harvest electrical energy from mechanical or radiant sources that are unrelated to the target analyte, and therefore require a separate recognition or electrochemical sensing layer to deliver analytical selectivity. This distinction, between platforms that are intrinsically self-powered by the sensing reaction itself and platforms that are power-assisted by an external physical energy source, is central to classifying the triboelectric and piezoelectric nanogenerator-based systems discussed next.

Within this framework, the terms power-assisted, self-charging, and hybrid describe distinct architectures. Power-assisted systems use energy from a separate harvester to operate or support the electrochemical sensing module; self-charging systems incorporate an energy-storage stage that is charged by the harvester and subsequently supports sensing or readout; and hybrid systems integrate multiple energy-harvesting, transduction, or sensing pathways within a single platform. TENG- and PENG-assisted electrochemical systems therefore should not automatically be classified as intrinsically self-powered biosensors. Because relatively few reported TENG/PENG platforms combine defined biochemical recognition with a dedicated electrochemical sensing mechanism, the following sections discuss selected examples that illustrate these architectures rather than treating TENG/PENG systems as a core class equivalent to intrinsically self-powered electrochemical biosensors.

#### 2.4.1 Triboelectric nanogenerator-assisted electrochemical biosensors

Triboelectric nanogenerators (TENGs) convert biomechanical motion, vibration, contact-separation, or sliding into electrical energy through triboelectrification and electrostatic induction (Sarode et al., 2026). In electrochemical biosensing, classification requires care: a TENG alone is not an electrochemical biosensor because it transduces mechanical energy rather than electrochemical recognition or redox reactions. However, TENGs are relevant when they power electrochemical sensing modules, wireless transmission, signal-processing circuits, or wearable biosensing systems. Figure 4 illustrates how a TENG can power electrochemical sensors (Khandelwal et al., 2022). In panel (a), mechanical motion or pressure activates the TENG, which converts mechanical energy into electricity. A power-management unit regulates this output and supplies a sensor probe or transducer, enabling chemical or biological detection without an external battery. Panel (b) presents the TENG as a general self-powered platform for chemical sensors detecting gases or molecules such as CO_2_, NO_2_, NH_3_, and ethanol, as well as biosensors targeting bacteria, glucose, dopamine, and lactate. Overall, the figure highlights TENGs as promising power sources for portable, wearable, and autonomous sensing systems.

**FIGURE 4:**
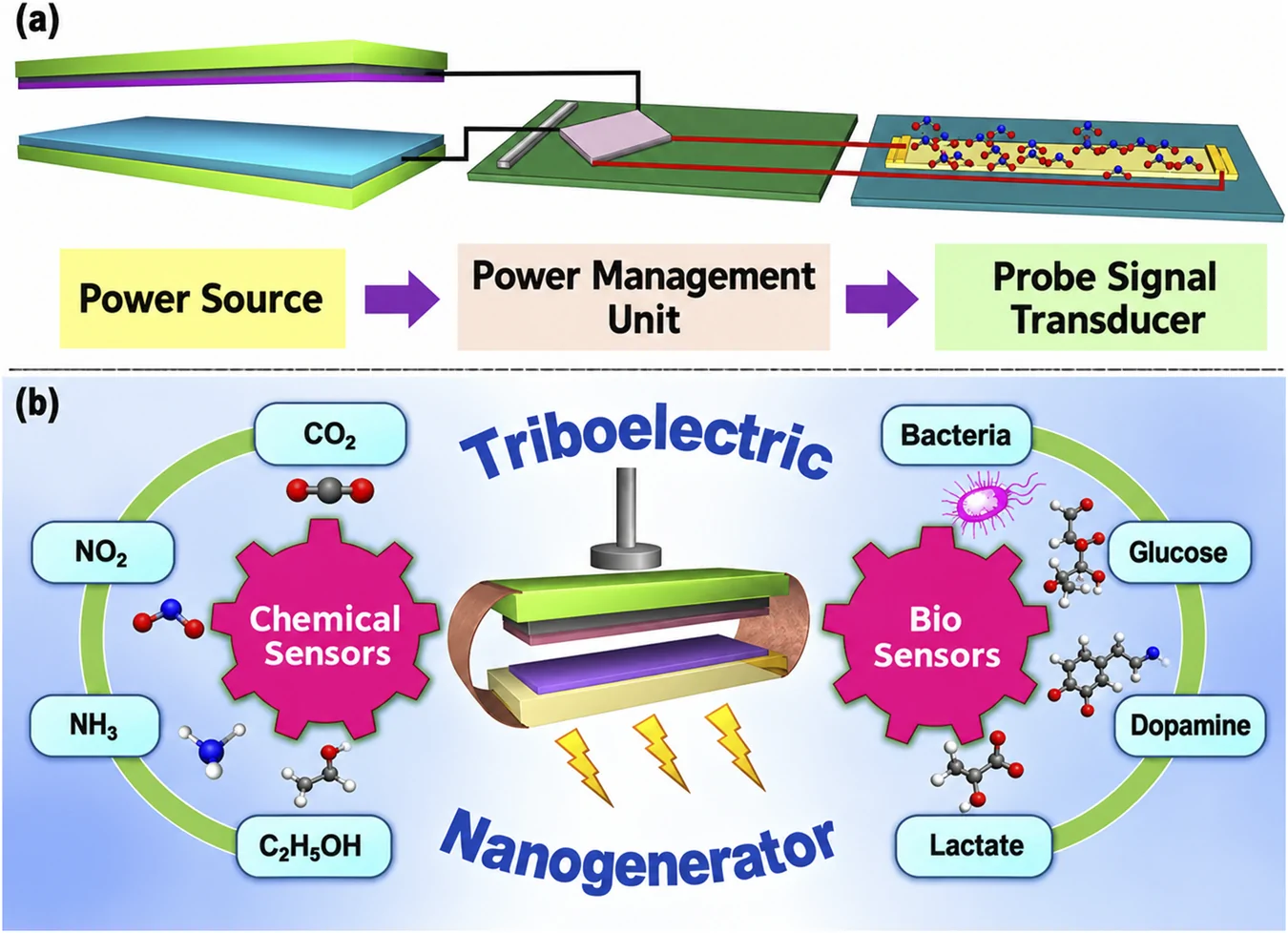
TENG-assisted sensing platform: **(a)** conversion of mechanical energy into electricity for sensor operation and **(b)** representative chemical-sensing and biosensing applications. Adapted from (Khandelwal et al., 2022) with permission.

Song et al. reviewed self-powered wearable biosensors and identified wearable energy harvesters that convert biomechanical motion into electricity as components for powering biosensors, signal-processing circuits, and wireless data transmission (Song et al., 2021). Similarly, Khandelwal et al. and Sarode et al. reviewed triboelectric nanogenerators as power sources for chemical sensors, biosensors, and physiological sensing highlighting their ability to supply energy for analyte detection (Khandelwal et al., 2022; Sarode et al., 2026). These studies support classifying TENG-based devices as energy-harvesting or power-assisted biosensing platforms, rather than electrochemical biosensors, unless they include an integrated electrochemical sensing interface.

A clear example is the wireless, battery-free wearable sweat sensor reported by Song et al. (2020). The system used a flexible printed-circuit-board-based freestanding TENG to harvest biomechanical energy from body motion. A power-management circuit then supplied energy to a multiplexed electrochemical sweat-sensing platform with wireless data transmission. Here, the electrochemical sensors performed biochemical detection, while the TENG enabled autonomous operation. Zhao et al. also demonstrated a self-powered electronic skin for real-time sweat Ca^2+^ monitoring and wireless transmission (Zhao et al., 2019). Although such systems are not classical three-electrode electrochemical biosensors, they are relevant to self-powered biosensing because they combine biochemical recognition, wearable sensing, self-generated output, and wireless readout.

Molecularly imprinted polymers (MIPs) have also been used in triboelectric biochemical sensing. Kanokpaka et al. developed a self-powered MIP-based triboelectric sensor for non-invasive lactate monitoring in sweat (Kanokpaka et al., 2022). In this device, the MIP selectively recognized lactate, while lactate adsorption altered the triboelectric output. This example shows that molecular-recognition layers, including MIPs, can be integrated with TENG-type devices for selective biochemical sensing. However, these devices are distinguished from conventional electrochemical biosensors because the measured signal is triboelectric, not amperometric, potentiometric, voltammetric, or impedimetric.

Thus, the main relevance of TENGs to electrochemical self-powered biosensors is their ability to serve as external or integrated power sources for electrochemical sensing and data transmission. In wearables, TENGs can harvest energy from walking, arm motion, joint movement, textile deformation, and other daily activities. The generated energy can be stored and used intermittently to power electrochemical measurement circuits, wireless modules, electrochromic displays, or smartphone-compatible readouts. Song et al. demonstrated this approach by using a freestanding TENG array for real-time sweat monitoring and wireless transmission during on-body testing (Song et al., 2020).

Despite these advantages, TENG-powered electrochemical biosensors face practical limitations. Their output depends strongly on motion amplitude and frequency, contact area, surface properties, humidity, load resistance, and packaging, all of which can vary during real-world wearable use (Pu et al., 2023). The electrochemical sensing signal must be separated from fluctuations in harvested energy. Reliable biochemical readout requires power-management circuits, energy storage, calibration strategies, and mechanically stable packaging.

TENG-powered electrochemical biosensors offer a credible route toward battery-free wearable and portable sensing. Their role is not to replace electrochemical recognition or signal generation, but to harvest mechanical energy for electrochemical sensing modules and wireless readout. Therefore, TENG-only pressure, motion, gait, respiration, or tactile sensors should not be classified as electrochemical biosensors. TENG-based devices are relevant to electrochemical self-powered biosensing only when they power an electrochemical sensor, support biochemical readout, or integrate a validated molecular-recognition interface for analyte detection and the following design rules in Box 4 may be applied to the development of TENGs.

BOX 4 **Design Rules for TENGs.**

1. Explicitly distinguish TENG-only mechanical energy harvesting from electrochemical biosensing by identifying the molecular-recognition and electrochemical-transduction mechanisms.
2. Match power-management circuitry (charge pumps, capacitors, duty cycling) to the electrochemical module’s actual power demand.
3. Evaluate mechanical durability and output stability under defined operating conditions. As an author-proposed benchmark rather than an established durability standard, at least 100 continuous mechanical cycles are recommended for preliminary evaluation of cyclic output stability, with mechanical amplitude and frequency specified and signal drift and/or RSD reported.
4. Decouple motion-driven output fluctuations from the target-dependent electrochemical signal during calibration.
5. Report energy-harvesting and analytical performance together, including appropriate TENG output metrics and electrochemical sensing metrics such as sensitivity, LOD, linear range, and response time.

#### 2.4.2 Piezoelectric nanogenerator-assisted electrochemical biosensors

Piezoelectric nanogenerators (PENGs) convert mechanical deformation, pressure, bending, vibration, and biomechanical motion into electrical signals through the piezoelectric effect. In wearable and portable sensing, they are attractive self-powered platforms because they can harvest energy from body motion, muscle activity, respiration, pulse, or tissue deformation (Zhu Q. et al., 2023; Sarode et al., 2026). In electrochemical biosensing, however, PENGs must be classified carefully. A PENG alone is not an electrochemical biosensor because its primary transduction is piezoelectric rather than redox-based, amperometric, potentiometric, voltammetric, or impedimetric. PENGs become relevant to electrochemical and biochemical sensing only when coupled with enzymatic reactions, molecular-recognition layers, or electrochemical sensing/readout units.

Recent reviews on self-powered wearable biosensors and piezoelectric/triboelectric chemical sensors describe PENGs as both energy-harvesting and sensing components in wearable healthcare systems (Song et al., 2021; Pu et al., 2023; Zhu Q. et al., 2023). These studies show that PENGs can support self-powered sensing, but biochemical selectivity still requires an added recognition layer, such as an enzyme, ion-sensitive film, antibody, aptamer, or molecularly imprinted polymer. Therefore, PENG-based systems are not grouped with enzymatic or microbial fuel cells in electrochemical self-powered biosensor classifications; instead, they are discussed as nanogenerator-assisted biosensing platforms that can complement electrochemical detection (Han et al., 2017).

Han et al. developed a self-powered wearable electronic skin for noninvasive perspiration analysis using an enzyme/ZnO nanoarray piezo-biosensing matrix (Han et al., 2017). In this device, ZnO nanoarrays acted as piezoelectric transducers, while enzymes supplied biochemical selectivity for sweat metabolites, including lactate, glucose, uric acid, and urea. The study demonstrates how enzymatic recognition can be coupled to piezoelectric self-powered output in wearables, but the mechanism is better described as piezo-biosensing rather than conventional electrochemical sensing. PENG-based biosensing has also been applied to lactate detection. Lin et al. reported a self-powered lactate sensor that coupled the piezoelectric effect with an enzymatic reaction for rapid tumor-sample analysis (Lin et al., 2024). The device used lactate oxidase-modified ZnO nanowire arrays, allowing lactate recognition to modulate the piezoelectric output. This work supports using enzyme-functionalized PENGs for clinically relevant metabolites, although the analytical signal remains piezoelectric rather than classical electrochemical.

For electrochemical biosensing, PENGs most practically serve as auxiliary energy harvesters. They can convert biomechanical inputs such as bending, pressure, body movement, or tissue deformation into electrical energy that can be stored in capacitors or used to power low-energy sensing circuits (Zheng et al., 2017). In this configuration, the electrochemical sensor performs biochemical detection, while the PENG powers signal acquisition, conditioning, display, or wireless transmission. This distinction is important because piezoelectric output depends strongly on motion amplitude, frequency, device placement, and mechanical coupling. Compared with TENGs, PENGs can offer compact designs, flexible-substrate integration, and sensitivity to small mechanical deformations. Materials such as ZnO, BaTiO_3_, PZT, PVDF, and piezoelectric composites are widely used for self-powered sensing. ZnO is especially relevant for biosensing because it is piezoelectric, semiconducting, biocompatible, and can be grown as high-surface-area nanowires or nanoarrays for enzyme immobilization (Pan et al., 2020). This explains the frequent use of enzyme/ZnO nanoarray architectures in self-powered biochemical sensing.

PENG-based self-powered biosensors are relevant to electrochemical biosensor reviews when they enable biochemical detection, support enzyme-coupled sensing, or power autonomous electrochemical systems. They are not described as electrochemical biosensors unless they include a genuine electrochemical sensing interface or readout. Their main current value lies in wearable self-powered biochemical sensing and energy harvesting for low-power biosensors. Future development should be guided by the following design rules and focused on improved power management, stable piezoelectric materials, durable biofunctionalization, motion-independent calibration, and clearer integration with electrochemical sensing modules in Box 5.

BOX 5 **Design Rules for PENGs.**

1. Incorporate a defined biochemical or chemical recognition interface, such as an enzyme, aptamer, antibody, molecularly imprinted polymer, or ion-selective membrane, when claiming analyte-specific biosensing. Piezoelectric output alone does not establish biochemical selectivity.
2. Select piezoelectric materials and device architectures that are compatible with the immobilization chemistry, mechanical requirements, and biocompatibility needs of the intended application.
3. Characterize the dependence of harvested output on mechanically relevant operating conditions. As an author-proposed benchmark rather than an established standard, evaluate output at three or more motion amplitudes or frequencies representative of the intended use, including a stationary baseline where applicable.
4. Distinguish motion-dependent energy harvesting from target-dependent analytical response and establish a calibration strategy that prevents variations in mechanical input from being interpreted as changes in analyte concentration.
5. Clearly identify the analytical transduction mechanism and the role of the PENG in the device architecture. Studies should distinguish whether the PENG supplies energy to an amperometric, voltammetric, potentiometric, impedimetric, or other electrochemical sensing module, or whether piezoelectric output itself constitutes the measured signal.

TENG- and PENG-based platforms are classified as energy-harvesting or power-assisted systems rather than electrochemical biosensors in their own right, distinguishing them from biofuel-cell platforms where energy generation and sensing are unified in the same device. Table 3 provides a comparative overview of the major electrochemical self-powered biosensing platforms discussed. Rather than classifying these systems solely by their energy source, the comparison integrates their molecular recognition elements, energy-conversion mechanisms, signal-generation strategies, principal strengths, key limitations, and representative applications. This framework highlights the fundamental distinction between intrinsically self-powered biofuel-cell platforms, in which energy generation and molecular recognition are directly coupled, and power-assisted nanogenerator-based systems, where externally harvested energy supports a separate electrochemical sensing interface. The comparison also emphasizes the analytical and translational trade-offs that should guide platform selection for wearable, implantable, environmental, and point-of-care diagnostic applications.

**TABLE 3:** Comparative overview of major electrochemical self-powered biosensing platforms.

Platform	Recognition/Energy element	Energy source	Signal generation mechanism	Major strength	Major limitation	Primary applications
Enzymatic biofuel cell (EBFC)	Isolated redox enzymes (e.g., glucose oxidase, lactate oxidase, alcohol dehydrogenase)	Biochemical fuel oxidation	Bioelectrocatalytic redox reactions	High molecular specificity; analyte can simultaneously serve as fuel and target; well suited for wearable and implantable metabolite sensing	Enzyme denaturation, mediator leaching, oxygen dependence, and low power density limit long-term operation	Glucose, lactate, cholesterol, ethanol, wearable and implantable biosensing
Microbial fuel cell (MFC)	Electroactive microbial biofilms	Microbial oxidation of organic substrates	Microbial extracellular electron transfer	Operates in complex environmental matrices and supports long-term autonomous monitoring	Slow response, lower molecular specificity, biofilm variability, and long start-up times	BOD, COD, toxicity, assimilable organic carbon, environmental monitoring
Organelle-based biofuel cell	Mitochondria, thylakoid membranes, chloroplast-derived systems	Biochemical or photosynthetic energy	Organelle-mediated bioelectrocatalysis or photobioelectrocatalysis	Complex biological responses and light-driven energy conversion that cannot be reproduced by isolated enzymes	Difficult organelle isolation, membrane fragility, photodamage, short operational lifetime, and limited analytical validation	Photosynthetic biosensing, toxicity monitoring, biohybrid energy systems
Triboelectric nanogenerator (TENG)-assisted platform	External electrochemical recognition layer (enzyme, antibody, aptamer, MIP, etc.)	Biomechanical motion, vibration, contact-separation	Triboelectrification and electrostatic induction	Enables battery-free wearable electrochemical sensing and wireless operation	Output depends on motion amplitude, frequency, contact area, humidity, and power-management circuitry; biochemical selectivity is provided by the recognition layer rather than the TENG itself	Wearable biosensors, sweat analysis, wireless health monitoring
Piezoelectric nanogenerator (PENG)-assisted platform	External electrochemical recognition layer (enzyme, antibody, aptamer, MIP, etc.)	Mechanical deformation, pressure, bending, biomechanical motion	Piezoelectric charge generation	Harvests biomechanical energy with simple device architectures suitable for wearable and implantable systems	Output depends on deformation and device placement; requires a separate biochemical recognition interface for analytical selectivity	Wearable health monitoring, implantable devices, self-powered electrochemical sensing

Table 4 provides a matched comparison of representative systems across analytical, energy-generation, operational-stability, and validation metrics. The table is divided into two linked panels for clarity, with Table 4 summarizing analytical performance and Table 5 reporting energy-generation, stability, and validation metrics for the same systems. Values are included only when explicitly reported for the referenced configuration; NR indicates not reported, and N/A indicates not applicable.

**TABLE 4:** Quantitative benchmarking of analytical performance in typical intrinsic and assisted systems.

Study/system	Target and recognition element	Sample matrix	Linear range	LOD	Sensitivity	Response time
Sekretaryova et al. (2014); intrinsic EBFC	Cholesterol; cholesterol oxidase bioanode/biocathode	human plasma	0.15–4.1 mM	1.4 µM	26.0 mA M^-1^ cm^-2^	NR for assembled device
Jeerapan et al. (2016); wearable EBFC	Glucose or lactate; glucose oxidase or lactate oxidase	Model solution; human sweat/on-body	NR	NR	NR	NR
Ouyang et al. (2016); affinity MIP–BFC	Glycoprotein; surface boronate-affinity MIP	Model/buffer system	1 ng mL^-1^–10 μg mL^-1^	1 ng mL^-1^	NR	NR
Hao et al. (2021); hybrid glucose/MnO_2_ EBFC	Glucose; glucose oxidase/CoPc/PBA bioanode	Buffer; grape juice; human serum	0.5–8 mM	NR	48.66 (stirred) and 32.12 (unstirred) μA cm^-2^ mM^-1^	NR
Tardy et al. (2021); MFC biosensor	BOD/biodegradable organics; electroactive microbial biofilm	Wastewater	59–660 mg L^-1^	∼20 mg L^-1^	NR	NR
Song et al. (2020); TENG-assisted electrochemical platform	pH; Na^+^: ionophore/PEDOT:PSS	human sweat	pH 4–8; Na^+^ 12.5–200 mM	NR	56.28 mV pH^-1^; 58.63 mV decade^−1^ for Na^+^	∼2 min for Na^+^ (50 → 200 mM at 2 μL min^-1^); pH response time NR
Han et al. (2017); PENG-assisted piezo-biosensor	Lactate, glucose, uric acid, and urea; enzyme/ZnO nanoarrays	Perspiration; on-body	NR	NR	NR	Real-time demonstration; numerical value NR

**TABLE 5:** Matched energy-generation, operational-stability, and validation metrics for the systems in Table 4.

Study/system	Energy source	OCV	Current/power density	Stability and operating duration	Real-sample/on-body/reference validation
Sekretaryova et al. (2014)	Cholesterol/O_2_	109 mV	Maximum power density: 11.4 μW cm^-2^ at 53 mV	OCV RSD 3.5%; maximum-power RSD 4.0% (n = 3); long-term duration NR	Platelet-poor plasma from five donors; comparison with a standard colorimetric cholesterol assay
Jeerapan et al. (2016)	Glucose or lactate/O_2_	0.44 V (glucose); 0.46 V (lactate)	Maximum power density: 160 μW cm^-2^ (glucose); 250 μW cm^-2^ (lactate)	Stable output after 100 cycles at 100% strain; operating duration NR	Sock-integrated, on-body human demonstration
Ouyang et al. (2016)	Glucose/O_2_ BFC; l	NR	NR	NR	Selective model-target/interference evaluation; authentic-sample validation NR
Hao et al. (2021)	Glucose/MnO_2_	NR	NR	Recovery 99.5%–101.2%; RSD <8%; operating duration NR	Grape juice and human serum recovery tests
Tardy et al. (2021)	Biodegradable wastewater organics	NR	Electrical BOD response reported; directly comparable power density NR	Study-specific operational tests; cross-platform duration metric NR	Wastewater containing readily and slowly biodegradable organics
Song et al. (2020)	Human motion via freestanding TENG	NR	0.94 mW maximum; 416 mW m^-2^ at 4.7 MΩ and 1.5 Hz	Minimal TENG-output decay after 20,000 cycles; sensors tested for 3 h and over >6 weeks; 60-min on-body demonstration	On-body human sweat monitoring with wireless transmission
Han et al. (2017)	Mechanical deformation via ZnO piezoelectric nanoarrays	NR	NR	Continuous runner demonstration; numerical duration and long-term drift NR	On-body, non-invasive perspiration analysis

Across the representative systems compared here, a recurring reporting imbalance is evident. EBFC studies generally provide quantitative bioelectrochemical outputs such as OCV or maximum power density, but analytical response time, long-term operational stability, and validation against reference methods are not consistently reported. Affinity-based systems may provide detailed analytical metrics such as linear range and LOD while offering less complete characterization of energy-generation performance and operational durability. Conversely, TENG/PENG-assisted systems often emphasize harvested electrical output, mechanical cycling stability, and wearable operation, while conventional analytical metrics such as LOD, sensitivity, and calibration independent of mechanical input may be less comprehensively characterized. These differences make direct cross-platform comparisons difficult and illustrate why analytical performance alone, or energy-generation performance alone, is insufficient to establish the maturity of a self-powered biosensor. More consistent reporting across analytical, energetic, operational, and validation dimensions is therefore needed to enable meaningful benchmarking and assessment of technological readiness.

## 3 Molecular recognition interfaces in electrochemical self-powered biosensors

Molecular recognition interfaces are essential in electrochemical self-powered biosensors because they determine whether the self-generated electrical response has analytical value. These devices can produce current, voltage, open-circuit voltage, power, photocurrent, stored charge, or wireless output, but they function as biosensors only when this signal is selectively linked to a biological or chemical target. Thus, molecular recognition is not simply a surface modification; it is the key interface connecting energy generation, electrochemical transduction, and diagnostic specificity (Slaughter and Kulkarni, 2015; Grattieri and Minteer, 2018).

This distinction matters because many self-powered platforms generate electricity without delivering selective biochemical information. In enzymatic biofuel-cell biosensors, enzymes can combine substrate recognition, fuel conversion, and signal generation. In microbial fuel-cell biosensors, electroactive biofilms act as living recognition and transduction layers that respond to biodegradable organic matter or toxic inhibition. In organelle-based systems, mitochondria or thylakoid membranes provide organized bioelectrocatalytic machinery. However, many clinically relevant targets, including proteins, nucleic acids, cytokines, cardiac markers, cancer antigens, viral antigens, cells, and exosomes cannot directly serve as fuels. These analytes therefore require additional recognition elements, such as antibodies, aptamers, DNA probes, molecularly imprinted polymers, ion-selective membranes, or nanozyme-assisted catalytic interfaces (Xue et al., 2024). Designing these interfaces is more challenging than in conventional electrochemical biosensors. The recognition layer must preserve or modulate the internally generated signal without adding excessive resistance, limiting diffusion, suppressing catalysis, or consuming more energy than the device produces. The central challenge is therefore to balance target selectivity with sufficient conductivity and permeability for efficient energy-signal coupling.

In affinity-based systems, the energy source, molecular-recognition element, and transduction interface are therefore functionally distinct. An antibody, aptamer, nucleic acid probe, or MIP captures the target; the biofuel cell or TENG/PENG-based energy-harvesting module provides or supports the required energy; and the electrode interface converts target-induced changes, such as interfacial blocking, conformational changes, hybridization, catalytic gating, or altered charge transfer, into a measurable analytical signal. This functional separation provides the framework used throughout Sections 3.4–3.7 to distinguish energy generation, molecular recognition, and analytical transduction.

### 3.1 Enzyme-based recognition interfaces

Enzyme-based interfaces are the most established recognition elements in electrochemical self-powered biosensors. In enzymatic biofuel cells, enzymes provide substrate selectivity while catalyzing redox reactions that convert biochemical energy into electrical output. Typical anodic enzymes include glucose oxidase, glucose dehydrogenase, lactate oxidase, lactate dehydrogenase, alcohol dehydrogenase, alcohol oxidase, and cholesterol oxidase, whereas bilirubin oxidase and laccase are commonly used at cathodes for oxygen reduction (Gupta and Krasnoslobodtsev, 2024). A major advantage of enzyme-based recognition is that the analyte can simultaneously serve as the target, fuel, and enzymatic substrate, particularly for glucose, lactate, ethanol, cholesterol, and related metabolites. This concept has been demonstrated in self-powered cholesterol detection in human plasma (Sekretaryova et al., 2014), ethanol sensing (Ruff et al., 2017), printed-circuit-board glucose detection (Gonzalez-Solino et al., 2021), and disposable enzymatic biofuel-cell glucose sensing (Morshed et al., 2023). These examples explain why enzyme interfaces remain the most mature strategy for metabolite-focused self-powered biosensors.

Enzyme interfaces are also well suited to wearable and implantable self-powered biosensors because endogenous fuels are naturally present in biological fluids. Sweat glucose and lactate, interstitial glucose, and blood metabolites can support both sensing and energy generation. Examples include stretchable textile biofuel cells for wearable self-powered sensing (Jeerapan et al., 2016), and a wireless battery-free sweat sensor powered by human motion (Song et al., 2020). Together, these studies show how enzyme-based interfaces combine biochemical selectivity with autonomous operation. Figure 5 shows a representative enzyme-based self-powered electrochemical biosensor based on an enzymatic glucose/oxygen fuel cell. In this system, glucose oxidase at the bioanode catalyzes glucose oxidation, while bilirubin oxidase at the biocathode catalyzes oxygen reduction, producing a self-generated current without an external power supply. This design illustrates how enzyme interfaces combine molecular selectivity, bioelectrocatalysis, and signal generation (Gonzalez-Solino et al., 2021). It also shows how metabolite concentration can be directly linked to current, voltage, or power output in self-powered biosensors.

**FIGURE 5:**
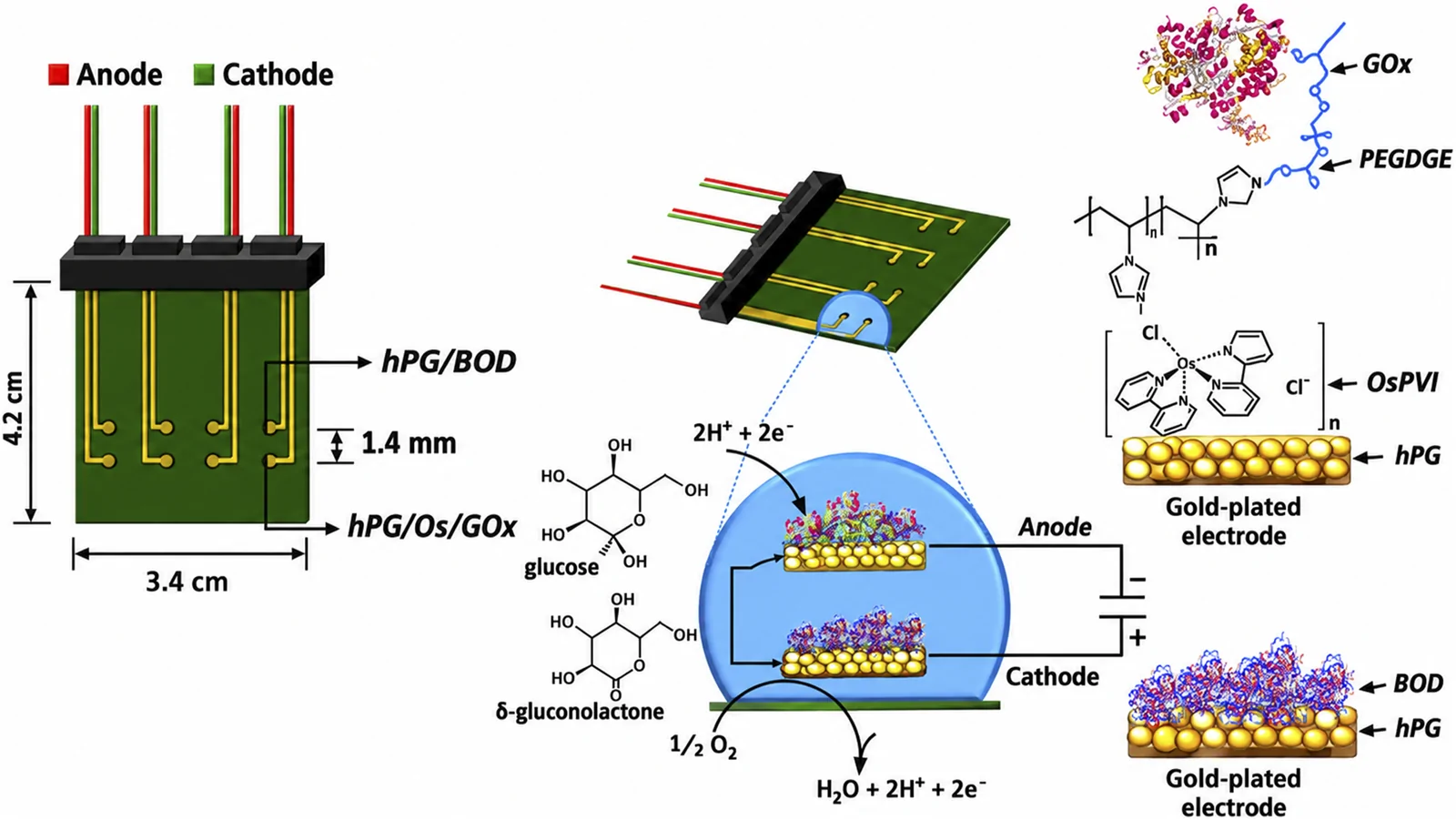
Schematic of the PCB-based EFC showing the GOx/Os/hPG/Au anode, SB/BOD/hPG/Au cathode, and corresponding electrochemical reactions. Adapted from (Gonzalez-Solino et al., 2021) with permission.

Despite their strengths, enzyme-based interfaces remain limited by pH, temperature, oxygen availability, substrate diffusion, immobilization chemistry, mediator stability, and sample-matrix effects. Enzymes can denature, leach from electrodes, or gradually lose activity during extended use. Many redox enzymes also have buried active sites, requiring mediators, conductive polymers, carbon nanomaterials, or redox hydrogels to improve electron transfer. Thus, enzyme interfaces must be optimized for operational stability, efficient charge transfer, antifouling performance, and compatibility with flexible or miniaturized electrodes.

### 3.2 Microbial biofilm interfaces

Microbial biofilms function as living recognition and bioelectrocatalytic interfaces in microbial fuel cell-based self-powered biosensors. Electroactive microorganisms oxidize organic substrates and transfer electrons to the anode through direct electron transfer, conductive pili, outer-membrane cytochromes, or soluble redox mediators. The resulting electrical output reflects microbial metabolism, substrate availability, and toxic inhibition, making these systems useful for biochemical oxygen demand, chemical oxygen demand, assimilable organic carbon, and toxicity monitoring (Sonawane et al., 2020). More recently, Xue et al. classified these biosensors into substrate-effect and microbial-activity-effect modes (Xue et al., 2024). In the first mode, current or voltage varies with biodegradable organic matter; in the second, toxic substances such as heavy metals, antibiotics, pesticides, phenols, or industrial pollutants inhibit microbial metabolism and reduce the electrical output.

A key advantage of microbial biofilm interfaces is their ability to operate in complex environmental samples and support long-term monitoring. Unlike isolated enzymes, microbial communities can adapt to wastewater, river water, and industrial effluents. However, they generally provide lower molecular specificity than enzyme-, antibody-, aptamer-, or MIP-based interfaces. Their response depends on microbial composition, biofilm maturity, pH, temperature, dissolved oxygen, nutrient availability, ionic strength, hydraulic conditions, electrode material, and external resistance. Thus, microbial biofilm interfaces are currently better suited to water-quality and toxicity monitoring than to precise clinical biomarker detection (Xue et al., 2024).

### 3.3 Organelle-based bioelectrocatalytic interfaces

Organelle-based bioelectrocatalytic interfaces are an emerging but important direction in electrochemical self-powered biosensing. As introduced in Section 2.3, these systems use functional subcellular components, including mitochondria and thylakoid membranes, whose organized electron-transfer chains and natural redox machinery can be coupled to electrodes for energy conversion and sensing. The mitochondria- and thylakoid-based biofuel cells described there illustrate the underlying bioelectrocatalytic mechanism; the recognition-relevant question is how these same components respond selectively to analytes, inhibitors, and environmental stressors once integrated into a sensing device. Figure 6 shows the thylakoid membrane-based photo-bioelectrochemical bioanode reported by Sarode and Slaughter (2025a) for self-powered light-driven bioelectronics (Sarode and Slaughter, 2025a). In this design, laser-induced graphene provides a porous conductive scaffold, PEDOT improves conductivity and biointerface stability, and immobilized thylakoid membranes act as the light-harvesting bioelectrocatalytic layer. Under illumination, the thylakoids generate photosynthetic electrons and produce a measurable photocurrent that can be paired with a cathode to form a photo-bioelectrochemical fuel cell. This example illustrates how natural photosynthetic membranes can be integrated with conductive nanomaterials to convert light into self-powered electrochemical output.

**FIGURE 6:**
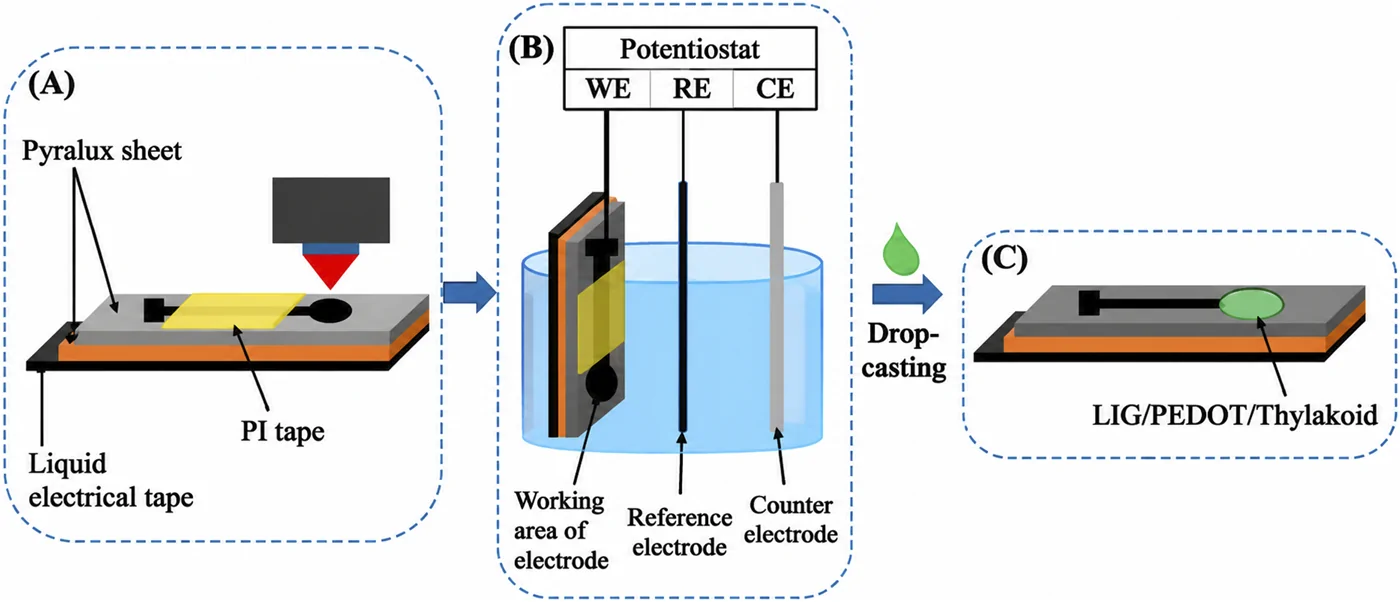
Schematic fabrication of the photoanode: **(A)** laser-induced graphene electrode preparation, **(B)** PEDOT electrodeposition on LIG, and **(C)** thylakoid drop-casting onto the LIG/PEDOT electrode (Sarode and Slaughter, 2025a).

As recognition interfaces, organelles can respond to substrates, inhibitors, oxidative stress, photosynthetic inhibitors, herbicides, light intensity, and environmental stressors. Mitochondria-based systems are relevant for studying respiratory inhibition, metabolic toxicity, and compounds that affect electron transport or membrane potential, whereas thylakoid- and photosystem-based interfaces are suited to photosynthetic inhibitor detection and environmental toxicity assessment. These same practical limitations, including organelle isolation, short lifetimes outside native environments, and membrane fragility, apply equally when organelles are used as recognition elements rather than energy sources alone. As recognition elements, then, organelles inherit the provisional status already established for them as energy sources (Masi et al., 2020).

### 3.4 Antibody-based recognition interfaces

Antibody-based interfaces enable self-powered detection of protein biomarkers, pathogens, toxins, and clinically relevant antigens. Antibody-antigen binding can modulate electrochemical or photoelectrochemical output by hindering electron transfer, increasing interfacial resistance, changing photocurrent generation, or limiting redox-species access to the electrode. This approach is particularly useful for non-fuel biomarkers that cannot directly produce energy (Grattieri and Minteer, 2018) Self-powered photoelectrochemical immunosensors provide clear examples of this strategy. Jiang et al. developed a dual-photoelectrode platform for cardiac troponin I detection (Jiang et al., 2022), while Caldas et al. reported a membraneless, zero-bias system using an anti-cTnI-modified photoanode (Caldas et al., 2023). Together, these studies demonstrate how antibody recognition can be integrated with self-powered photoelectrochemical platforms for clinically important cardiac biomarkers.

Antibody interfaces are also applicable to cancer biomarkers, inflammatory proteins, viral antigens, bacterial toxins, and food-safety targets. In self-powered devices, antibodies can be immobilized on the bioanode, biocathode, photoelectrode, or a separate recognition electrode, depending on whether target binding is designed to block electron transfer, inhibit catalysis, alter photocurrent, or initiate amplification. However, antibodies are expensive, biologically fragile, and sensitive to temperature, pH, dehydration, and storage. They are also prone to nonspecific adsorption and limited regeneration. For autonomous diagnostics, they are therefore best paired with stable immobilization, antifouling protection, and reliable calibration strategies (Gupta and Krasnoslobodtsev, 2024).

Despite these limitations, antibodies remain important recognition elements for selective detection of protein and antigen targets. Their use in self-powered platforms, however, differs fundamentally from enzymes that simultaneously provide molecular recognition and fuel conversion. Antibodies generally function as non-fuel recognition layers, with target binding modulating electron transfer, catalytic activity, or another downstream signal-generation process. Alternative receptors such as aptamers and MIPs can offer advantages in synthetic tunability, storage stability, regeneration, or manufacturing, although these benefits are application dependent and do not make any receptor class universally superior. Consequently, antibodies remain the clinical benchmark for protein biomarker specificity, but they are the most fragile and highest-cost recognition element, in contrast to the storage stability of MIPs or the synthetic tunability of aptamers. In self-powered platforms specifically, antibody-based interfaces are essentially always non-fuel recognition layers rather than energy sources, so their future development depends less on the antibody itself than on pairing stable immobilization and antifouling protection with a photoelectrochemical or biofuel-cell platform capable of sustaining the coupled energy-generation reaction.

### 3.5 Aptamer-based recognition interfaces

Aptamers are synthetic single-stranded DNA or RNA receptors that recognize targets through sequence-specific folding. They offer useful alternatives to antibodies because they are chemically synthesizable, readily functionalized, sometimes regenerable, and easily integrated with electrochemical nanomaterials (Cheng et al., 2009). In self-powered electrochemical biosensors, aptamer–target binding can alter current, voltage, power, or stored charge by changing electron-transfer distance, steric hindrance, electrode accessibility, mediator diffusion, or catalytic activity (Radi and Abd-Ellatief, 2021). A representative example is a carbon nanotube-gold nanoparticle self-powered electrochemical aptasensor for myoglobin detection (Zhu Y. et al., 2023). In this platform, a glucose oxidase bioanode supplied power, while an aptamer-functionalized biocathode selectively recognized myoglobin. Target binding altered the self-powered electrochemical output, enabling protein biomarker detection in complex biological samples (Figure 7).

**FIGURE 7:**
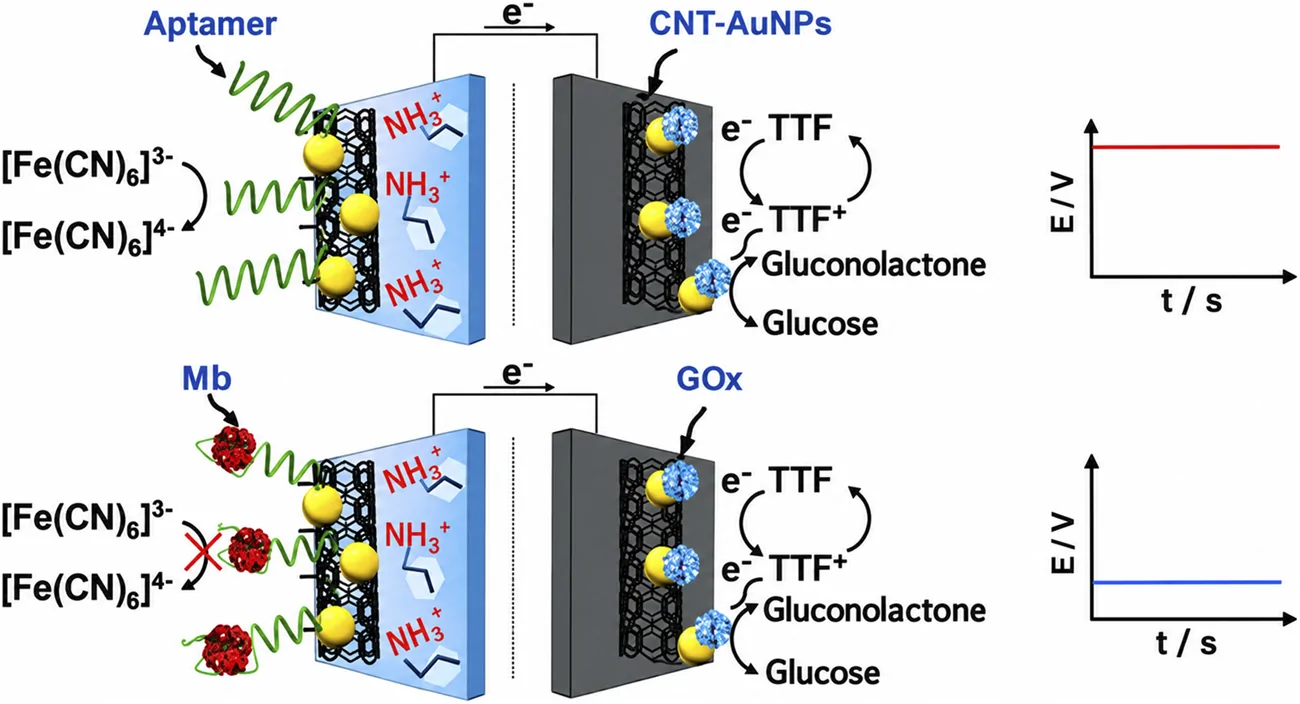
Self-powered EBFC aptasensor for myoglobin detection. The CNT-AuNP-GOx bioanode generates the self-powered signal through glucose oxidation, while the CNT-AuNP-aptamer biocathode selectively recognizes myoglobin. Myoglobin binding hinders redox-probe electron transfer at the biocathode, decreasing the open-circuit voltage (E_OCV_) and enabling ultrasensitive detection (Zhu Y. et al., 2023).

Aptamer interfaces are well suited for detecting proteins, small molecules, cells, toxins, and disease biomarkers. They integrate readily with nanostructured electrodes, including carbon nanotubes, graphene, gold nanoparticles, and MXenes, which enhance receptor loading and electron-transfer coupling. Their performance, however, depends on aptamer affinity, folding stability, surface density, spacer design, ionic strength, nonspecific adsorption, and sample-matrix effects. In self-powered platforms, the aptamer layer must be positioned so that target binding produces a measurable change in fuel-cell output, photocurrent, or stored charge. Compared with antibodies, aptamers offer chemical synthesis, batch-to-batch consistency, and in some formats regeneration, but their affinity and folding stability are generally more sensitive to ionic strength and surface density than an antibody-antigen interaction. Antibodies and aptamers should therefore be regarded as complementary recognition strategies rather than universally superior alternatives. Like antibodies, aptamers generally function as non-fuel recognition elements and must be coupled to an appropriate energy-generation and transduction architecture. Future development should prioritize aptamer sequences selected or engineered for stable recognition and signal transduction directly in the intended sample matrix rather than relying primarily on affinity optimized under idealized buffer conditions.

### 3.6 DNA probe and nucleic-acid recognition interfaces

DNA probes offer programmable recognition interfaces for detecting miRNAs, viral RNA, bacterial DNA, circulating tumor DNA, single-nucleotide polymorphisms, and other genetic biomarkers, as established in electrochemical nucleic-acid biosensing (Bai et al., 2023). Because nucleic acids are not typical electrochemical fuels, their sequence-specific hybridization must regulate a separately generated signal from an enzymatic biofuel cell or self-powered photoelectrochemical platform. This principle has been demonstrated in EBFC-based SNP detection, DNA-walker-assisted microRNA assays, capacitor-coupled microRNA bioassays, and photo-driven self-powered microRNA sensing (Grattieri and Minteer, 2018).

Direct hybridization is straightforward but often inadequate for low-abundance targets, especially short miRNAs and rare mutant ctDNA, where sequence similarity, low copy number, and complex biological matrices demand amplification (Zhang et al., 2022). In self-powered electrochemical biosensors, this challenge has been addressed by integrating nucleic-acid amplification with enzymatic biofuel-cell outputs or capacitor-assisted readout. DNA walker systems, for example, have been coupled with enzymatic biofuel cells and capacitors for ultrasensitive microRNA detection (Wang et al., 2023; Yang et al., 2023). These target-activated walkers repeatedly traverse surface-bound DNA tracks, generating multiple hybridization or strand-displacement events that amplify the electrical response. DNAzyme walkers have likewise enabled self-powered dual-mode electrochemical/electrochemiluminescent sensing of miRNA-21 (Du et al., 2023). Catalytic hairpin assembly (CHA) and hybridization chain reaction (HCR) have also been used to control enzymatic loading on biofuel-cell electrodes, converting microRNA recognition into amplified self-powered signals, as shown for miRNA-141 detection using a glucose/O_2_ biofuel-cell platform (Zhao et al., 2023). CHA- and HCR-assisted systems have also been reported for dual microRNA assays, including miRNA-21 and miRNA-155 in serum (Wang et al., 2023). Collectively, DNA walkers, DNAzyme walkers, CHA, and HCR convert weak hybridization events into measurable changes in current, voltage, stored charge, or capacitor-assisted readout in self-powered electrochemical biosensors.

Nucleic-acid recognition can also control self-powered photoelectrochemical output. In these systems, target-induced hybridization or DNA conformational changes modulate photosensitizer coupling, charge separation, electron-donor accessibility, or interfacial recombination. Gai et al. (2020) reported a photoelectrochemical enzymatic fuel-cell-based self-powered biosensor for microRNA detection using DNA conformation-controlled co-sensitization, achieving ultrasensitive detection in human serum (Gai et al., 2020). Yu et al. (2021) later developed a photo-driven self-powered microRNA biosensor based on metal-organic-framework-controlled ascorbic acid release, showing that target-triggered molecular release can regulate photo-biofuel-cell output (Yu et al., 2021). Genetic variants can also be detected by coupling allele-selective nucleic-acid recognition with enzymatic biofuel-cell output; for example, Gu et al. (2019) combined DNA amplification with an enzymatic biofuel-cell-based self-powered biosensor for ultrasensitive single-nucleotide polymorphism detection (Gu et al., 2019). Taken together, these photoelectrochemical implementations show that nucleic-acid recognition can regulate a light-driven, rather than purely bioelectrocatalytic, self-powered signal, extending the same amplification logic developed for biofuel-cell-coupled DNA sensing to a second transduction mechanism.

Unlike enzymes or organelles, DNA probes cannot generate energy on their own, so nucleic-acid recognition always depends on a separately powered biofuel cell or photoelectrochemical platform; this is a structural difference from EBFC- and MFC-based sensing, where recognition and energy generation occur in the same reaction. Relative to antibodies and aptamers, DNA probes offer the greatest programmability and the widest range of compatible amplification chemistries (DNA walkers, DNAzyme walkers, CHA, HCR), but this same reliance on amplification and multistep sample handling makes them the least autonomous of the affinity-based recognition interfaces discussed in this section.

Despite their sensitivity, nucleic-acid recognition interfaces remain limited by hairpin leakage, nuclease degradation, nonspecific adsorption, slow hybridization, electrode-surface crowding, and matrix-dependent probe folding. Multistep incubation, washing, and reagent addition can further reduce autonomy in self-powered electrochemical biosensors. Future designs should emphasize one-pot amplification, nuclease-resistant probes, antifouling interfaces, internal references, microfluidic reagent handling, and mismatch-selective recognition. Capacitor-assisted amplification is also distinguished from continuous power generation, since capacitors enhance instantaneous readout but do not necessarily increase the intrinsic biofuel-cell power.

### 3.7 Molecularly imprinted polymer interfaces

Molecularly imprinted polymers offer stable artificial recognition interfaces for electrochemical self-powered biosensors. They are formed by polymerizing functional monomers around a target template, which is then removed to generate cavities that match the target in size, shape, and chemical functionality (Ben Moussa et al., 2025; Lahcen and Slaughter, 2025). Compared with enzymes, antibodies, and aptamers, MIPs are low-cost, chemically robust, storage-stable, and compatible with harsh conditions, making them attractive for autonomous sensing platforms (Lahcen et al., 2022). In electrochemical self-powered biosensors, MIPs usually do not serve as the energy source; rather, they function as selective interfacial layers that modulate the self-generated signal. Target rebinding within the imprinted cavities can impede electron transfer, restrict access of redox probes or substrates, increase interfacial resistance, alter charge transport, or reduce photocurrent generation. Thus, MIPs are especially useful for non-fuel targets that cannot directly drive biofuel-cell reactions, including glycoproteins, pesticides, toxins, metabolites, cancer-related molecules, and environmental contaminants.

Indeed, several electrochemical self-powered biosensors demonstrate this role. Ouyang et al. (2016) developed a surface protein-imprinted, biofuel-cell-based self-powered sensor for glycoprotein detection, in which the MIP selectively recognized horseradish peroxidase as a model glycoprotein while the biofuel cell produced the electrochemical output (Figure 8) (Ouyang et al., 2016). In contrast, Wang et al. (2022) reported a hollow-channel paper analytical device powered by a glucose/air biofuel cell for self-powered pesticide detection. In this system, MIPs prepared with 2,4-dichlorophenoxyacetic acid as the template controlled the output through competitive recognition (Wang et al., 2022). This example shows that MIPs can be directly linked to enzymatic biofuel-cell outputs, including current, voltage, power, and stored-charge readouts.

**FIGURE 8:**
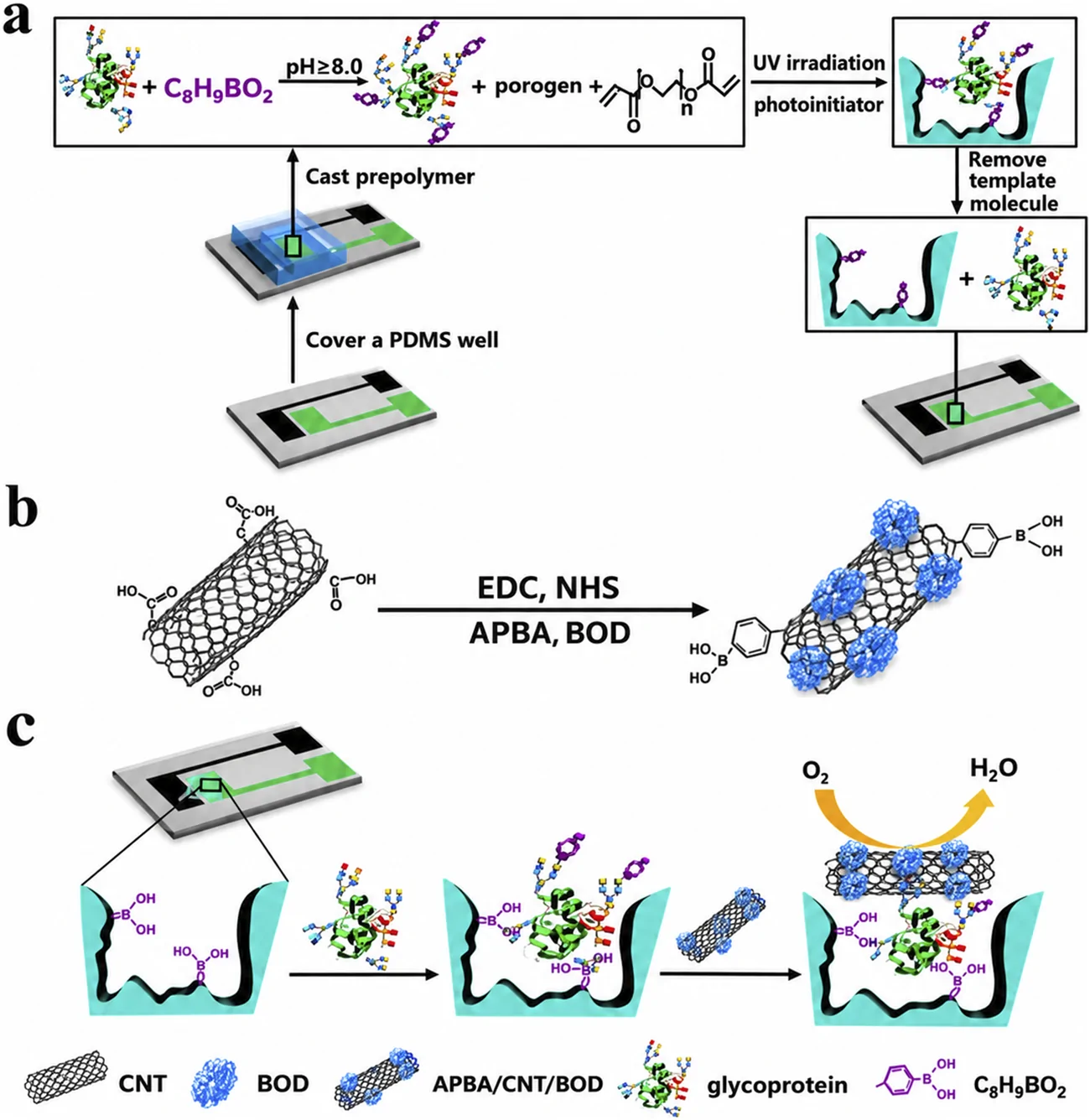
**(a)** Fabrication of the boronate-affinity MIP-modified electrode, **(b)** preparation of APBA/CNT/BOD nanocomposites, and **(c)** MIP-electrode mechanism for glycoprotein recognition and oxygen reduction (Ouyang et al., 2016).

MIPs have also been incorporated into self-powered photoelectrochemical and photocatalytic fuel-cell-assisted sensors. Jin et al. (2021) developed a photocatalytic fuel-cell-assisted molecularly imprinted self-powered sensor for aflatoxin B1, in which the MIP provided target selectivity while the photocatalytic fuel cell generated the analytical signal (Jin et al., 2021). In addition, Lu et al. (2023) further reported a dual-photoelectrode MIP-based photoelectrochemical sensor for sialic acid detection (Lu et al., 2023). Collectively, these examples demonstrate that MIPs generalize across both transduction mechanisms: target rebinding modulates a bioelectrocatalytic current in Ouyang et al. and Wang et al., and a photoinduced charge-transfer signal in Jin et al. and Lu et al., with the imprinted layer performing the same selective-blocking function in either case.

Despite these benefits, MIP interfaces are still limited by incomplete template removal, nonspecific adsorption, slow rebinding, and reduced selectivity in complex matrices. Imprinting large biomolecules is particularly difficult because proteins or cells can denature, become trapped, or diffuse slowly through the polymer network. Surface imprinting, epitope imprinting, boronate-affinity approaches, and thin electropolymerized MIP films can improve binding-site accessibility, mass transport, and interfacial electron transfer. However, successful integration into self-powered systems requires careful control of polymer thickness, permeability, template removal, cavity accessibility, and nonspecific adsorption because an excessively thick or poorly conductive recognition layer can impede electron transfer or substrate transport and reduce the internally generated analytical signal. MIPs offer advantages including synthetic robustness, storage stability, tunable recognition chemistry, and potential regeneration, but their performance remains strongly dependent on monomer-template interactions, target size, fabrication conditions, and sample matrix. Their suitability should therefore be evaluated at the device level based on selectivity, response time, stability, regeneration, cost, and compatibility with the intended energy-generation and transduction architecture rather than on receptor type alone.

Overall, MIPs expand electrochemical self-powered biosensors to non-fuel targets by converting selective rebinding into changes in current, voltage, power, photocurrent, or electron transfer. Future work will improve film control, template removal, antifouling performance, mass transport, and coupling between recognition and signal generation.

### 3.8 Nanozyme and biomimetic catalytic interfaces

Nanozymes and biomimetic catalysts are gaining prominence in electrochemical self-powered biosensors because they can supplement or replace natural enzymes, enhance catalytic stability, and sustain robust signal generation (Huang et al., 2019; Gu et al., 2022). Compared with enzymes, nanozymes typically better tolerate pH changes, temperature variation, storage, and chemical stress, making them well suited for autonomous sensing platforms In biofuel cell-based self-powered biosensors, enzyme/nanomaterial hybrids, light-driven systems, and enzyme-free nanocatalytic strategies have been reviewed as effective routes to improve durability, stability, and sensing performance (Gu et al., 2022). In enzymatic biofuel cells, nanozymes or catalytic nanomaterials can enhance either the bioanode or biocathode while maintaining self-powered electrochemical readout. For example, Hao et al. (2021) developed a mediator-free self-powered glucose biosensor based on a hybrid glucose/MnO_2_ enzymatic biofuel cell. The glucose oxidase bioanode produced the glucose-dependent signal, while the MnO_2_ capacitive cathode increased output and eliminated the need for external redox mediators (Hao et al., 2021).

Nanozyme and biomimetic catalysts also support enzyme-free self-powered electrochemical sensing (Hasan et al., 2018; Banerjee et al., 2022; Lei et al., 2025). Hasan et al. demonstrated that hybrid bioelectrode architectures incorporating colloidal platinum and bilirubin oxidase can improve electron-transfer efficiency and cathodic bioelectrocatalysis, providing an effective strategy for enhancing the performance of enzymatic biofuel cells (Hasan et al., 2018). Similarly, Banerjee et al. demonstrated the feasibility of a fully abiotic, skin-conformal biofuel cell capable of simultaneously harvesting biochemical energy and sensing glucose without relying on enzyme-based catalysis, highlighting the potential of abiotic electrocatalysts for stable and wearable self-powered biosensing. Whereas Lei et al. (2025) demonstrated a non-enzymatic biofuel-cell-based glucose biosensor using Au nanocluster/Pd nanocube heterostructures as glucose oxidase-like anodic catalysts and Fe_3_C@C-Fe single-atom catalysts as cathodic catalysts (Lei et al., 2025). These designs show how nanozyme-like electrocatalysts can replace natural enzymes while improving operational robustness in glucose sensing.

Biomimetic catalysts are also useful in fuel-cell-based self-powered electrochemical sensors for hydrogen peroxide. Zhang et al. (2024) reported a self-powered H_2_O_2_ sensor using a fuel-cell architecture with FePc and FePc/graphene cathode catalysts and a Ni anode. In this case, H_2_O_2_ served as the chemical fuel, and the resulting fuel-cell output functioned as the sensing signal (Zhang et al., 2024). Nanozyme and biomimetic catalytic interfaces can increase catalytic activity, enable mediator-free operation, improve anodic or cathodic reactions, and amplify signals in electrochemical self-powered biosensors. However, they often provide lower molecular selectivity than enzymes, antibodies, aptamers, or MIPs, and their responses may be influenced by pH, ionic strength, dissolved oxygen, redox interferents, surface poisoning, aggregation, or leaching.

Compared with enzymes and affinity receptors, nanozymes and biomimetic catalysts generally emphasize catalytic activity and physicochemical robustness rather than highly specific molecular recognition and may exhibit greater stability than natural enzymes under some environmental conditions. However, their activity, selectivity, and stability depend strongly on material composition, morphology, surface chemistry, target, and operating environment, and any claimed performance advantages should therefore be validated for the specific device and application. Relative to MIPs, nanozymes and biomimetic catalysts provide catalytic turnover rather than selective binding through imprinted cavities. Their most effective role in self-powered electrochemical biosensors may therefore be as catalytic interfaces, electron-transfer enhancers, signal-amplification components, or enzyme alternatives, with additional recognition elements incorporated when greater molecular selectivity is required.

### 3.9 Ion-selective and chemical recognition interfaces

Ion-selective membranes and chemically responsive layers broaden self-powered wearable electrochemical biosensors beyond oxidizable metabolites to include electrolytes and physicochemical sweat markers. Key sweat parameters, including pH, Na^+^, K^+^, Ca^2+^, NH_4_^+^, and Cl^−^, provide non-invasive insight into hydration, osmotic balance, electrolyte homeostasis, muscle fatigue, and physiological stress (Bandodkar and Wang, 2014). Because sweat is increasingly viewed as a physiologically informative biofluid, the demand for reliable multi-ion sensing is accelerating the shift of wearable devices from laboratory prototypes toward practical platforms (Yang et al., 2026). Solid-contact potentiometry is the main strategy for sweat-ion detection. In this approach, an ion-selective electrode converts target-ion activity into an interfacial potential. Solid-contact ion-selective electrodes are particularly suitable for wearables because they do not require an internal filling solution and can be fabricated on flexible or printable substrates. Conducting polymers, carbon nanotubes, reduced graphene oxide, and MXenes are commonly used as solid contacts because they support stable ion-to-electron transduction, low charge-transfer resistance, and high double-layer or redox capacitance (Gao et al., 2024). A recent review identifies nanomaterial-based solid contacts as essential for accurate sweat monitoring of Na^+^, K^+^, Ca^2+^, Mg^2+^, NH_4_^+^, and Cl^−^, with applications in sports monitoring and early detection of dehydration, fatigue, and muscle spasms (Fathy and Bühlmann, 2025).

Because Na^+^, K^+^, Ca^2+^, and Cl^−^ are not biofuel-cell fuels, they do not directly generate power through anodic oxidation. Their detection therefore relies on changes in potential, conductivity, triboelectric output, or capacitance. Two main integration strategies are used. The first, and more established, uses a separate energy harvester to power ion-selective electrodes and wireless electronics. For example, Song et al. (2020) developed a battery-free wearable FTENG platform that generated ∼416 mW m^-2^ from body motion and enabled multiplexed sweat pH and Na^+^ sensing with Bluetooth transmission during on-body tests (Song et al., 2020). Min et al. (2023a) further advanced this approach using a flexible quasi-two-dimensional perovskite solar-cell module with >31% indoor power-conversion efficiency to power simultaneous monitoring of glucose, pH, Na^+^, sweat rate, and skin temperature for more than 12 h across indoor and outdoor activities (Min et al., 2023a). The second strategy allows the ion-recognition event to directly modulate the harvested electrical signal. Zhao et al. (2019) reported a self-powered electronic skin for real-time Ca^2+^ monitoring in sweat, where coupled triboelectrification and enzymatic reactions produced a triboelectric current proportional to Ca^2+^ concentration while also supporting wireless data transmission (Zhao et al., 2019). At the component level, flexible potentiometric platforms combining Na_0.44_MnO_2_, polyaniline, and K_2_Co[Fe(CN)_6_] enabled simultaneous Na^+^, K^+^, and pH sensing with near-Nernstian sensitivities and real-time smartphone readout via Wi-Fi during on-body experiments (Jalal et al., 2024). These examples show that ion-selective sensing has so far been realized either by dedicating a separate harvester to the ion-recognition electrode (Song et al., 2020; Min et al., 2023a; Jalal et al., 2024) or by letting the ion-binding event directly gate the harvested signal (Zhao et al., 2023), but in every case the ion itself never functions as the fuel, distinguishing this recognition class fundamentally from the enzyme- and organelle-based platforms discussed earlier.

Ion-selective interfaces also support matrix correction in multiparametric wearable systems. Measuring pH, ionic strength, temperature, and sweat rate alongside metabolites can improve enzymatic-sensor accuracy because these variables affect enzyme activity, mediator redox behavior, and electrode potential independently of the target concentration (Min et al., 2023b). These interfaces are the only recognition class discussed here that targets simple ions rather than a biomolecule, and unlike enzymes, antibodies, aptamers, or MIPs, they are essentially never coupled to a biofuel-cell energy source directly; every self-powered ion-sensing example described here relies on a separate triboelectric, piezoelectric, or photovoltaic harvester rather than the sensing reaction itself. This places ion-selective platforms structurally closer to the TENG- and PENG-assisted systems than to the intrinsically self-powered EBFC and MFC platforms.

Despite these advances, wearable ion-selective interfaces remain vulnerable to potential drift, reference-electrode instability, ionophore leaching, membrane dehydration, biofouling, and interference from competing ions (Gao N. et al., 2025). Unresolved issues such as signal drift, limited on-body calibration, and uncertain sweat-to-blood correlations continue to hinder clinical translation (Bariya et al., 2018; Davis et al., 2024). In self-powered wearables, these problems are intensified because harvested energy from triboelectric generators or biofuel cells can fluctuate with body motion, fuel availability, internal resistance, and power-management efficiency, increasing variability in signal conditioning and calibration (Song et al., 2020; Davis et al., 2024; Gupta and Krasnoslobodtsev, 2024). Future platforms should combine durable SC-ISE membranes, miniaturized solid-state reference electrodes, antifouling coatings, microfluidic sweat handling, and adaptive calibration that accounts for temperature, sweat rate, and matrix composition.

Ion-selective interfaces detect ionic species through selective ion recognition and ion-to-electron transduction rather than fuel oxidation. Their integration requirements therefore differ fundamentally from those of enzymatic biofuel-cell, affinity-based, and microbial systems. In self-powered architectures, the ion-selective interface primarily provides recognition and analytical transduction, while autonomous operation generally requires a separate energy-generation or energy-storage component because the target ion does not itself serve as the electrochemical fuel.

Overall, ion-selective and chemical recognition interfaces broaden self-powered electrochemical biosensing to continuous, battery-free electrolyte monitoring while providing essential matrix information to improve the reliability of enzyme-based wearable sensors.

### 3.10 Interface engineering strategies

Interface engineering is central to converting molecular recognition into measurable self-powered electrochemical signals. In these platforms, the recognition layer must selectively bind the target while maintaining efficient electron transfer, mass transport, catalytic activity, and long-term stability. This challenge is greater than in conventional electrochemical biosensors because energy conversion, signal generation, and analytical detection often occur within the same device. Consequently, carbon nanomaterials, metal nanoparticles, conductive and redox polymers, hydrogels, antifouling coatings, and porous electrodes are commonly used to strengthen the coupling between recognition events and current, voltage, power, photocurrent, or stored-charge readouts.

Carbon nanomaterials, including carbon nanotubes, graphene, reduced graphene oxide, carbon cloth, carbon paper, porous carbon, and laser-induced graphene, are widely used because they offer high surface area, strong conductivity, mechanical flexibility, and suitable surfaces for enzyme or receptor immobilization. For instance, carbon nanotube-gold nanoparticle interfaces have enhanced aptamer loading and electron-transfer communication in self-powered aptasensors for myoglobin detection (Zhu Y. et al., 2023). Similarly, printed circuit board-based glucose/oxygen enzymatic fuel cells used porous gold-modified electrodes and osmium redox polymer wiring to enable glucose oxidase-based self-powered glucose sensing (Gonzalez-Solino et al., 2021). In photo-bioelectrochemical systems, laser-induced graphene provides a porous conductive scaffold for biohybrid interfaces, such as PEDOT/thylakoid photoanodes, improving light-driven electron collection and self-powered output (Sarode and Slaughter, 2025a).

Metal nanoparticles, particularly gold nanoparticles, are valuable interface-engineering components because they enhance conductivity, increase effective surface area and receptor loading, and provide versatile surface chemistry for biomolecule immobilization. In electrochemical biosensors, AuNPs can improve electron transfer between immobilized biomolecules and the electrode surface while supporting stable biomolecule attachment (Pingarrón et al., 2008). Au–S chemistry is especially useful for anchoring thiolated aptamers and DNA probes on gold surfaces, making it widely used in electrochemical aptasensor and DNA-sensor design (Oberhaus et al., 2020; Zhu Y. et al., 2023).

Conductive and redox polymers enhance charge transport and enzyme-electrode communication in self-powered electrochemical systems. Conductive polymers such as PEDOT, polypyrrole, and polyaniline provide chemically versatile matrices for immobilizing biological components and forming electropolymerized recognition films, including MIPs (Ramanavičius et al., 2022). For example, PEDOT-functionalized screen-printed carbon electrodes have been used to immobilize glucose oxidase and facilitate mediator-free glucose detection, demonstrating the potential of conductive polymers to improve enzyme-electrode coupling in third-generation biosensors (Chen et al., 2022). In contrast, redox polymers containing osmium complexes or ferrocene groups can electrically wire enzymes whose active sites are poorly coupled to the electrode, promoting mediated electron transfer and increasing enzymatic biofuel cell output (Gracia and Mecerreyes, 2013; Xiao et al., 2019). Osmium-based redox polymers, for example, have been used to wire enzymes in glucose/O_2_ biofuel cells for self-powered glucose detection (Gonzalez-Solino et al., 2021). Despite these advantages, polymer swelling, mediator or redox-component leaching, restricted substrate or oxygen transport, and long-term interfacial instability remain important limitations.

Hydrogels and porous electrodes help preserve biological activity while supporting the transport of fuels, analytes, oxygen, ions, and redox species. Hydrogels create hydrated microenvironments for enzymes and other recognition elements, whereas porous electrodes increase electrochemically active surface area and biomolecule loading (Kuis et al., 2020). These advantages are especially useful in wearable and implantable enzymatic biofuel cells (Xiao et al., 2019; Kuis et al., 2020; Fredj et al., 2025). However, overly thick or poorly conductive hydrogel layers can increase diffusion resistance and weaken electron transfer, reducing the generated current or voltage.

Antifouling protection is critical for self-powered biosensor operation in complex matrices such as sweat, saliva, serum, urine, wastewater, and food extracts. Proteins, cells, metabolites, and other sample components can block recognition sites and disrupt electron transfer or mass transport. Hydrophilic polymers, zwitterionic coatings, hydrogels, selective membranes, and related layers can reduce nonspecific adsorption and improve signal stability (Campuzano et al., 2019; Sabaté del Río et al., 2019). In self-powered systems, however, these protective layers must remain thin and permeable enough to avoid suppressing the internally generated signal (Kuis et al., 2020). Thus, interface engineering is best understood as integrated system design rather than simple material modification. Electrode architecture, recognition chemistry, catalytic layers, redox wiring, mass transport, and antifouling protection must be co-optimized according to the energy source and signal-output mechanism. This integrated approach is essential for maintaining selectivity while enabling efficient self-powered electrochemical transduction.

### 3.11 Challenges and design rules for molecular recognition interfaces

Molecular recognition interfaces determine whether the electrical signal from a self-powered electrochemical platform has analytical value. Yet they also create many of the barriers that keep self-powered biosensors at the proof-of-concept stage. Unlike conventional electrochemical biosensors, which can use an external potential to overcome some interfacial constraints, self-powered systems rely on internally generated current, voltage, power, photocurrent, or stored charge. As a result, receptor instability, biofouling, nonspecific adsorption, slow mass transport, and poor signal coupling can directly compromise both analytical performance and power-generation efficiency. Receptor stability is a key challenge. Enzymes can denature, lose activity, or leach from electrodes during prolonged wearable or implantable use (Kuis et al., 2020; Gupta and Krasnoslobodtsev, 2024; Fredj et al., 2025). Microbial biofilms can adapt to complex matrices, but their response is strongly influenced by biofilm age, microbial composition, pH, temperature, nutrient availability, and dissolved oxygen (Hao et al., 2020; Xue et al., 2024). Organelle-based interfaces, including mitochondria and thylakoid membranes, offer promising bioelectrocatalytic activity but are limited by fragility, short lifetime, and inefficient electron transfer (Arechederra and Minteer, 2008; Ryu et al., 2018). Other receptors, such as antibodies, aptamers, DNA probes, MIPs, nanozymes, and ion-selective membranes, address some of these issues but introduce trade-offs in immobilization, regeneration, selectivity, and matrix compatibility.

Biofouling and matrix effects are equally important barriers. Complex samples such as sweat, saliva, serum, urine, wastewater, and food extracts contain salts, proteins, cells, metabolites, surfactants, and redox-active interferents that can foul the sensing surface or alter the local electrochemical environment. In wearable devices, sweat rate, evaporation, skin contamination, temperature variation, and motion artifacts can further reduce signal reliability (Song et al., 2020; Gao N. et al., 2025). In microbial and environmental systems, pH, ionic strength, organic load, oxygen levels, and toxic compounds can affect both microbial metabolism and electrode response. These effects are particularly problematic in self-powered biosensors because matrix-driven signal drift can be misinterpreted as a target-dependent output change.

Another major challenge is weak coupling between recognition and signal generation. Fuel-like targets such as glucose, lactate, ethanol, or cholesterol can directly regulate biofuel-cell output, whereas many biomarkers, including proteins, nucleic acids, toxins, cancer markers, and inflammatory mediators, must instead modulate electron transfer, mediator access, interfacial resistance, enzyme loading, photocurrent, or stored charge. Poor coupling can produce devices that generate power but lack sensitivity, or selective receptors that do not yield measurable self-powered signals. Receptor chemistry, electrode architecture, catalytic layers, mass transport, and readout format must therefore be co-designed. Interfaces must remain selective, permeable, conductive, and stable, since thick films, dense MIP layers, compact hydrogels, crowded DNA/aptamer surfaces, or protective membranes can improve capture or stability but hinder diffusion and electron transfer. Key design rules include matching the receptor to the target class, directly linking recognition to a measurable electrical output, improving antifouling protection without suppressing signal generation, and validating performance early in real samples rather than relying only on buffer calibration.

Recognition-interface performance should therefore be evaluated using both analytical and self-powered metrics. Analytical characterization should include sensitivity, linear range, detection limit, response time, selectivity, reproducibility, recovery, and operational stability, while energetic characterization should include appropriate measures such as open-circuit voltage, current or power density, internal resistance, charging capability, and signal stability, depending on the device architecture. Validation in authentic samples is particularly important because biofouling, nonspecific adsorption, matrix composition, environmental conditions, and motion can affect both molecular recognition and energy generation. Key design priorities are therefore to match the receptor to the target class, directly couple recognition to a measurable electrical output, provide antifouling protection without suppressing signal generation, and validate performance under conditions representative of the intended application. Future progress will depend on recognition interfaces that are selective, stable, antifouling, manufacturable, and efficiently coupled to self-powered electrochemical signal generation.

## 4 Translational barriers and future design rules

Despite significant progress in enzymatic and microbial biofuel-cell biosensors, other intrinsically self-powered electrochemical platforms, and selected TENG/PENG-assisted power-assisted, self-charging, and hybrid architectures, electrochemical self-powered biosensors continue to face major barriers to translation. These include power management, interfacial stability, real-sample reliability, scalable manufacturing, and limited standardization of performance evaluation and reporting. Biofuel cell-based devices are especially attractive because they can function simultaneously as the power source and sensing platform, reducing the need for external potentiostats or batteries (Slaughter and Kulkarni, 2015; Grattieri and Minteer, 2018; Hao et al., 2020). However, their power output often remains too low for continuous wireless communication, signal processing, or display operation, particularly in wearable and implantable formats. This limitation has driven the use of charge-pump circuits, capacitors, supercapacitors, intermittent duty cycling, and passive or low-power readout approaches such as NFC/RFID. Although motion-powered wearable systems show that harvested energy can support electrochemical sweat sensing and wireless transmission, careful power budgeting is still essential for autonomous operation (Song et al., 2020; Li et al., 2025).

Long-term operational stability is another major challenge. Enzymatic systems can be limited by enzyme denaturation, cofactor depletion, mediator leaching, oxygen dependence, and gradual loss of bioelectrocatalytic activity, whereas microbial fuel cell biosensors are affected by biofilm maturation, microbial-community shifts, environmental fluctuations, and recovery after toxic exposure (Kuis et al., 2020; Gupta and Krasnoslobodtsev, 2024; Xue et al., 2024). Organelle-based platforms, including mitochondria- and thylakoid-derived bioelectrodes, add further complexity because isolated biological membranes are fragile and sensitive to light, oxygen, hydration, and electron-transfer conditions (Liu et al., 2023). These limitations are amplified in real samples such as sweat, saliva, urine, blood, wastewater, and food extracts, where fouling, nonspecific adsorption, pH shifts, ionic-strength variation, fluctuating analyte flux, and redox-active interferents can distort the self-powered signal. In wearable devices, sweat-rate changes, evaporation, and mechanical deformation can further disrupt mass transport and electrode-biointerface integrity (Sivasubramanian et al., 2026; Galstyan et al., 2024).

Translational validation should therefore progress from buffer or model-system testing toward spiked matrices, authentic samples, on-body, *in vivo*, or field evaluation where appropriate, comparison with established reference methods, and assessment of long-term operation. These categories provide a descriptive validation framework rather than a formal maturity scale because analytical performance, energy-generation capability, and operational stability may progress independently. A device may, for example, demonstrate strong analytical performance in authentic samples while remaining insufficiently characterized for long-term autonomous operation.

More broadly, translation requires consideration of material reproducibility, biocompatibility, system integration, scalable manufacturing, and validation under clinically or environmentally relevant conditions in addition to analytical sensitivity (Chaudhary et al., 2026). These requirements are particularly important for autonomous and bio-integrated platforms intended for point-of-care, wearable, implantable, environmental, and One Health applications. Device architectures must therefore be designed not only for analytical performance but also for reproducible fabrication, integration of recognition and energy-generation components, biocompatibility where required, and scalable manufacturing.

Mechanically powered wearable systems introduce an additional validation requirement because variations in harvested energy can be mistaken for analyte-dependent signal changes. In TENG/PENG-assisted electrochemical systems, analyte-dependent responses should therefore be distinguished from changes caused by motion amplitude, frequency, or mechanical coupling. Appropriate controls can include measurements at fixed analyte concentrations across defined mechanical conditions, together with analyte calibration under controlled and reproducible mechanical inputs. Such testing is necessary to demonstrate that changes in the reported analytical signal originate primarily from the target-dependent sensing mechanism rather than variations in energy harvesting.

Redox mediators, metallic nanomaterials, quantum dots, encapsulation layers, flexible substrates and adhesives, must be assessed not only for electrochemical performance but also for skin compatibility, implant safety, leaching, sterilization, and long-term exposure. Scalable methods such as screen printing, inkjet printing, laser writing, printed circuit boards, and roll-to-roll processing can lower cost and improve batch reproducibility, but they must maintain biointerface activity and electrode-to-electrode consistency. Sustainability is also important for disposable point-of-care and wearable devices, making biodegradable substrates, soft polymer electronics, reduced battery dependence, and lower electronic waste increasingly relevant design goals (Li et al., 2025).

Future work should move away from isolated proof-of-concept studies and toward integrated analytical-energy systems. First, reports must clearly distinguish directly self-powered electrochemical biosensors from externally biased, energy-assisted, nanogenerator-powered, and self-charging hybrid platforms. Second, match recognition interfaces to the target class: enzymes for fuel-like metabolites, microbial biofilms for BOD/COD and toxicity monitoring, antibodies and aptamers for proteins, DNA probes for nucleic acids, MIPs for robust artificial recognition, nanozymes for catalytic amplification, and ion-selective membranes for electrolytes. Third, energy generation, molecular recognition, electron transfer, sample handling, storage, and readout electronics are best co-engineered rather than optimized separately. Finally, studies should report both analytical metrics such as sensitivity, limit of detection, selectivity, linear range, response time, reproducibility, and real-sample recovery, and self-powered metrics, including open-circuit voltage, short-circuit current, current density, power density, internal resistance, charging time, duty cycle, load stability, and readout power demand. Standardized reporting will enable meaningful comparison across platforms and accelerate translation toward wearable, implantable, point-of-care, and environmental monitoring applications.

## 5 Conclusion

Electrochemical self-powered biosensors offer a promising route toward autonomous diagnostics by integrating energy conversion, molecular recognition, and signal generation within a single platform. Enzymatic and microbial biofuel cells, abiotic catalytic systems, photo-bioelectrochemical devices, and nanogenerator-assisted architectures have enabled battery-free detection of metabolites, biomarkers, pathogens, nucleic acids, and environmental contaminants. Their performance depends not only on energy harvesting but also on how efficiently recognition events are translated into current, voltage, power, photocurrent, or stored-charge outputs. Interface engineering is therefore central to device performance. Carbon nanomaterials, metal nanoparticles, conductive and redox polymers, hydrogels, porous electrodes, selective membranes, and antifouling coatings can enhance receptor loading, electron transfer, mass transport, catalytic activity, and stability. However, greater interfacial complexity does not necessarily improve performance, as thick films, excessive porosity, mediator leaching, biofouling, and transport limitations can weaken the self-generated signal. Advancing these platforms toward practical applications will require more consistent reporting of both analytical and energy-generation performance, assessment of long-term operational stability, validation in realistic sample matrices and operating environments, motion-controlled calibration for wearable systems, integrated power management, reproducible and scalable manufacturing, and benchmarking against appropriate reference methods. Incorporating these considerations within the proposed design-rule framework can help move electrochemical self-powered biosensors beyond proof-of-concept demonstrations toward reliable systems for wearable, implantable, point-of-care, and environmental monitoring applications.

Future studies should emphasize integrated device design, durable recognition interfaces, controlled fluid handling, reliable calibration, low-power electronics, and validation in real biological and environmental samples. Clear distinctions between truly self-powered sensors and externally assisted systems are also needed for meaningful comparison. By co-designing energy generation, molecular recognition, interfacial transport, and signal readout, electrochemical self-powered biosensors can advance from proof-of-concept demonstrations toward reliable wearable, implantable, point-of-care, and decentralized monitoring technologies. Looking forward, the field is likely to evolve from analyte-powered proof-of-concept devices toward integrated autonomous microsystems that combine molecular recognition, energy harvesting, low-power electronics, wireless communication, artificial-intelligence-assisted analysis, and scalable manufacturing. Realizing this vision will depend less on isolated gains in sensitivity or power output than on treating recognition chemistry, energy conversion, and readout electronics as a single co-engineered system from the outset.
